# The oleic/palmitic acid imbalance in exosomes isolated from NAFLD patients induces necroptosis of liver cells via the elongase-6/RIP-1 pathway

**DOI:** 10.1038/s41419-023-06161-9

**Published:** 2023-09-26

**Authors:** Maria Principia Scavo, Roberto Negro, Valentina Arrè, Nicoletta Depalo, Livianna Carrieri, Federica Rizzi, Rita Mastrogiacomo, Grazia Serino, Maria Notarnicola, Valentina De Nunzio, Tamara Lippolis, Pasqua Letizia Pesole, Sergio Coletta, Raffaele Armentano, Maria Lucia Curri, Gianluigi Giannelli

**Affiliations:** 1 Personalized Medicine Laboratory, National Institute of Gastroenterology “S. de Bellis”, IRCCS Research Hospital, Via Turi 27, Castellana Grotte, 70013 Bari, Italy; 2 Institute for Chemical-Physical Processes (IPCF)-CNR SS Bari, Via Orabona 4, 70125 Bari, Italy; 3 Dipartimento di Chimica, Università degli Studi di Bari Aldo Moro, Via Orabona 4, 70125 Bari, Italy; 4 Experimental Immunopathology Laboratory, National Institute of Gastroenterology “S. de Bellis” IRCCS Research Hospital, Via Turi 27, Castellana Grotte, 70013 Bari, Italy; 5 Laboratory of Nutritional Biochemistry, National Institute of Gastroenterology “S. de Bellis”, IRCCS Research Hospital, Via Turi 27, Castellana Grotte, 70013 Bari, Italy; 6 Department of Pathology, “S. de Bellis” IRCCS Research Hospital, Via Turi 27, Castellana Grotte, 70013 Bari, Italy; 7 Scientific Direction, National Institute of Gastroenterology “S. de Bellis” IRCCS Research Hospital, Via Turi 27, Castellana Grotte, 70013 Bari, Italy

**Keywords:** Metabolic disorders, Necroptosis

## Abstract

Excessive toxic lipid accumulation in hepatocytes underlies the development of non-alcoholic fatty liver disease (NAFLD), phenotypically characterized by necrosis and steato-fibrosis, whose molecular mechanism is not yet fully understood. Patients with NAFLD display an imbalanced palmitic (PA) to oleic acid (OA) ratio. Moreover, increasing experimental evidence points out a relevant involvement of the exosomal content in disease progression. Aim of the study was to highlight the PA/OA imbalance within circulating exosomes, the subsequent intracellular alterations, and the impact on NALFD. Liver cells were challenged with exosomes isolated from both healthy subjects and NAFLD patients. The exosomal PA/OA ratio was artificially modified, and biological effects were evaluated. A NAFLD-derived exosomal PA/OA imbalance impacts liver cell cycle and cell viability. OA-modified NAFLD-derived exosomes restored cellular viability and proliferation, whereas the inclusion of PA into healthy subjects-derived exosomes negatively affected cell viability. Moreover, while OA reduced the phosphorylation and activation of the necroptosis marker, Receptor-interacting protein 1 (phospho-RIP-1), PA induced the opposite outcome, alongside increased levels of stress fibers, such as vimentin and fibronectin. Administration of NAFLD-derived exosomes led to increased expression of Elongase 6 (ELOVL6), Stearoyl-CoA desaturase 1 (SCD1), Tumor necrosis factor α (TNF-α), Mixed-lineage-kinase-domain-like-protein (MLKL) and RIP-1 in the hepatocytes, comparable to mRNA levels in the hepatocytes of NAFLD patients reported in the Gene Expression Omnibus (GEO) database. Genetic and pharmacological abrogation of ELOVL6 elicited a reduced expression of downstream molecules TNF-α, phospho-RIP-1, and phospho-MLKL upon administration of NAFLD-derived exosomes. Lastly, mice fed with high-fat diet exhibited higher phospho-RIP-1 than mice fed with control diet. Targeting the Elongase 6–RIP-1 signaling pathway offers a novel therapeutic approach for the treatment of the NALFD-induced exosomal PA/OA imbalance.

## Background

Steatosis is characterized by the accumulation of triglycerides in the liver and by an increment of esterified free fatty acids (FAs) in the hepatocytes [1], that is correlated with worsening fibrosis, leading to a severe rearrangement of the liver architecture [2]. An altered metabolic pathway of FAs was found in NAFLD patients compared to healthy subjects, suggesting potentially hazardous levels of these molecules [3]. Patients with NAFLD have significantly higher levels of total saturated FAs, such as palmitic acid (PA) versus monounsaturated fatty acids (MUFAs), such as oleic acid, OA) [4]. A high value of the dietary PA:MUFA ratio may accelerate the pathophysiological conditions of NAFLD patients, suggesting that an excessive imbalance of the two components rather than the PA level itself has detrimental health effects [5]. Exosomes are vesicles with a diameter ranging between 30 and 150 nm, that contain bioactive molecules. Recently, the extracellular vesicles (EVs) and their cargo, such as PA [6] have been implicated in the pathogenesis and progression of NAFLD. EVs derived from adipose tissue [6] have been reported to carry different types of molecules secreted from the adipocytes, named adipokines [7] which modulate the activity of specific distal recipient cells, including hepatocytes [6]. Recently, it has been reported that the elongation of very long chain fatty acids family member 6 enzyme (ELOVL6), which regulates the elongation of saturated FAs and monounsaturated C12-16 fatty acids (pMUFAs), might play a role in this context by attenuating the PA-mediated hepatotoxicity.

In addition, while several groups have suggested an involvement of necroptosis pathway in NAFLD onset and progression [8, 9], others have proposed that it may be driven by a pyroptosis form of caspase-dependent cell death [10, 11]. Hence, elucidating the underlying cell death pathway might foster the development of new targeted pharmacological therapies. Necroptosis, or programmable necrosis, is a mechanism of cell death that mimics apoptosis and necrosis, whose pathway is now fairly well-defined. Upon the engagement of tumor necrosis factor receptor 1 by the TNFα ligand, the pathway activates the phosphorylation and activation of receptor-interacting serine/threonine-protein kinase-1 (RIP-1) and RIP-3, forming a complex called a ripoptosome [12], which, in turn, co-migrates with the pseudokinase mixed lineage kinase domain-like protein (MLKL) to the nucleus, thus preceding the necroptotic-mediated cell death event [13]. Inflammasomes are multicomponent complexes consisting of intracellular receptors and scaffold proteins, which interpose the first line of defense against invading pathogens [14, 15]. The inflammasome pathway, ultimately, leads to the activation of caspase-1 (CASP1) and gasdermin D (GSDMD), a plasma membrane pore forming protein, leading to mature cytokines release into the blood stream, and pyroptosis [16, 17]. Recently, Zeng et al. demonstrated that increased levels of PA at the expense of OA are responsible for inflammasome and GSDMD activation, leading to pyroptosis-mediated cell death [12]. However, Akazawa and Nakao, as well as other groups, independently reported that high PA levels induce phospho-RIP-1-mediated necroptosis [10, 18, 19] with a promotion of mitochondrial ROS that allows the auto-phosphorylation of RIP-1 [20, 21]. In addition, although several studies have demonstrated the toxicity of exosomes carrying PA, no studies have yet been conducted on the balance between PA and OA within the exosomes, and hence on their ratio, as the crucial player in the cell death event, rather than the individual exosome fatty level. The aim of this study is to investigate whether an altered exosome balance between PA and OA is responsible for hepatocyte death in NAFLD patients.

## Materials and methods

### Patients

This trial was conducted at the National Institute of Digestive Diseases Laboratory of Epidemiology and Biostatistics, I.R.C.C.S. IRCCS “S. de Bellis”, Castellana Grotte, Italy. A detailed description of the characteristics of the study are described in the Patients section of the Supporting Information.

### Animals

Nine-week-old C57BL/6J wild-type male mice were obtained from Charles River Laboratories Italia (Calco, Italy). The “Guide for the Care and Use of Laboratory Animals” for all experiments involving animals was followed and the animal-use methods were communicated to and approved by the Italian Ministry of Health. The fully animal treatment protocol is reported in the Supporting Information.

### Exosome isolation, loading with fatty acids, and characterization

Plasma specimens from all individuals (60 NAFLD patients and 20 healthy subjects) were processed to extract exosomes according to the procedure reported by Scavo et al. and according to the criteria specified in MISEV 2018 [22, 23].

For their loading with PA or OA, PA (0.0641 g), or OA (0.0706 g) was dissolved in 100% ethanol (2.5 mL), at a final concentration of 100 mM, and then mixed with 20% (% w/w) fatty acid-free BSA (Sigma Aldrich, 22.5 mL), in phosphate-buffered saline (PBS) at 50 °C for 1 h, reaching a final concentration of 10 mM. All stock solutions were stored at −20 °C. Exosomes isolated from plasma (500 µL) of healthy subjects and NAFLD-affected patients were treated with 250 μL of PA and OA respectively, in BSA solution. Exosomes were recovered as pellet by ultracentrifugation, while the supernatants were kept for lipidomic analysis. The FA containing exosomes (hybrid exosomes) were finally extruded (Mini Extruder, Avanti Polar Lipids) and dispersed in ultrapure water (250 µL) for dynamic light scattering analysis, ζ-potential measurements, and transmission electronic on microscopy characterization [24], or in PBS (pH 7.2) for cellular administration.

### Lipidomic assay of exosomes

The FA profile in membranes of exosomes and hybrid exosomes of all subjects was evaluated using the Moilanen method [25], a modified form of the method used by Folch [26]. Each sample of exosomes was thawed to room temperature. FAs were hydrolyzed from phospholipids of exosome membranes by adding 450 µL of an acidified salt solution (H_2_SO_4_ 2 × 10^−4^ M, NaCl 0.1%). Then, 2.25 mL of a chloroform: methanol (2:1, v/v) mixture were added (Sigma-Aldrich, Milan, Italy) and the samples were mixed and centrifuged at 1000 × *g* for 20 min. The lower layer, containing FAs, was gently removed, placed in a clean tube and dried with a centrifugal evaporator (Bio-Rad, Milan, Italy). Then, FA methyl esters (FAME) were prepared by adding 250 µL of toluene (Sigma-Aldrich, Milan, Italy cod.24511) and 750 µL of BF_3_•MeOH 14% (Sigma-Aldrich, Milan, Italy cod 9005-64-5) and incubating for 2 h at 80 °C. In each sample, 1.250 mL of 5% aqueous sodium chloride solution and 250 µL of toluene were added and the resulting mixtures were centrifuged at 470 × *g* for 10 min. The FAME, being the upper layer, was collected, transferred into vials and analyzed.

### Fatty acids gas chromatography quantification

FAs quantification was performed by gas chromatography with an auto-sampler, a split/split-less injector, FID detector and a hydrogen gas generator (Thermo Fisher Scientific, Milan, Italy), as reported in the Supporting Information.

### Cells proliferation assay (MTS)

Hepa-RG cells, a reliable human hepatocyte cell line retaining several characteristics of human hepatocytes, were treated with exosomes isolated from plasma of all NAFLD patients and healthy subjects, before and after loading with PA or OA, and cell viability was evaluated by MTS assay. Details on cell proliferation experiments are reported in the Supporting Information.

### Propidium iodide (PI) fluorescence and Annexin-5 cell death assay

Cell death was induced to test the cytotoxic activity of exosomes derived from all healthy subjects and NAFLD patients; particularly propidium iodide (PI) was used as a dye which penetrates only damaged cellular membranes, with the consequent intercalation into double-stranded DNA, which ultimately creates an amplification effect of the fluorescence, during the early cellular death. Extended protocol is reported in the Supporting Information.

### Cell cycle assay

The cell cycle was observed using the Cell-Clock™ Cell Cycle Assay (Biocolor Life Science Assay, Carrickfergus County Antrim, UK cod. 264.7), a live cell detection and measurement system that is able to monitor the cell cycle phases during in vitro culture. This type of assay uses a redox type of dye which can be up taken by the cycling cells. Following dye uptake and incubation, distinct color changes occur within cells; such color changes are associated to cells in the G0–G1 (yellow staining), S (green staining), G2 and M (dark green/blue staining) phases. Cells were seeded into 24-well plates at a density of 2 × 10^4^ cells/well in HBM culture medium (Lonza cod 185389); after 24 h, the cells were treated with exosomes derived from all healthy donors and all subjects affected by NAFLD or with FA-loaded exosomes. The exosomes total protein content concentration was kept constant at 20 μg/μL. For PA-loaded hybrid exosomes from healthy subjects and OA-loaded hybrid exosomes from NAFLD patients, the PA (Sigma-Aldrich cod P0500) and OA (Sigma-Aldrich cod O1008) concentration was 10 and 13.5 µM, respectively. After 12 h, the conditioned medium was removed and replaced with 500 μL of fresh medium. Subsequently, 150 μL of cell-clock dye reagent were added on the center of each well, followed by incubation for 1 h at 37 °C. The dye reagent was gently removed from each well and, after washing with fresh medium, replaced with 200 μL of fresh medium. Images of live cells were acquired and analyzed by means of an Eclipse Ti2 Nikon confocal microscope at 20× magnification, and all experiments were conducted in triplicate. The calculation of phase ratios was obtained by computer software analysis (Image J) of the digitized images of photomicrographs.

### Immunofluorescence for imaging of fibers and markers of necroptosis and pyroptosis

Immunofluorescence analysis was performed on the Hepa-RG cell line, seeded into sterile chamber slides (Thermo-Fisher) at a density of 1 × 10^4^ cells/well and treated as reported in the Supporting Information using exosomes derived from 20 patients affected by NAFLD and 10 healthy subjects.

### Protein extraction and quantification from nuclei and cytoplasm derived from cell lines

Western blotting analysis was performed on the total protein content extracted from Hepa-RG cells treated with exosomes or hybrid exosomes derived from 20 patients affected by NAFLD with severe steatosis and 10 healthy subjects, according to the protocols reported in the Supporting Information.

### Silencing of ELOVL6

Hepa-RG cells were seeded into sterile six-well culture plates at a density of 2 × 10^5^ cells/well. After 24 h, cells were silenced for 48 h using the si-PORT-NeoFX transfection agent (Thermo Fisher Scientific cod. AM4510) and silencer select ELOVL6-siRNA (Thermo Fisher Scientific cod. AM51331), according to the manufacturer’s instructions. Details on cell proliferation experiments are reported in the Supporting Information.

### Inhibition of RIP-1 in Hepa-RG cell line by using Necrostatin-1

The inhibition of RIP-1 phosphorylation was conducted using the RIP-1 inhibitor named Necrostatin-1 (Sigma Aldrich cod N9037) in the Hepa-RG cells treated with exosomes derived from the patients affected by NAFLD, compared with untreated cells and cells treated with exosomes derived from healthy subjects (see the extended protocol in the Supporting Information).

### ROS detection in Hepa-RG cell line

Detection of intracellular free radicals was conducted by loading Hepa-RG cells plated in six wells plate with 4 μM of 2,7-dichlorofluorescein diacetate (DCFH-DA Sigma Aldrich cod D6883-50MG) in medium HBM phenol red-free, at 37 °C for 30 min [27]. Then, the cells were treated with exosomes derived from NAFLD patients or exosomes derived from healthy subjects; cells treated only with DCFH-DA or with 100 μM of H_2_O_2_ were the negative and positive controls (CTR), respectively. After incubation, the culture medium was removed and cells were rinsed three times with PBS, lysed with Tris-HCl (Sigma Aldrich cod T6455) 10 mM/NaCl (Sigma Aldrich cod S 7653) 150 mM/Triton X-100(Sigma Aldrich cod T8787) 0.5%, pH 7.5 and then centrifuged at 10.000 × *g*, at 4 °C for 10 min. Supernatants were collected and their spectrofluorometric analysis was performed at 525 nm under excitation at 485 nm. Results were normalized to total proteins and Reactive Oxygen Species (ROS) production was expressed as a relative percentage of photoluminescence intensity compared to the negative control.

### Immunohistochemical investigation on mice liver tissue

Nine-week-old C57BL/6J wild-type male mice were obtained from Charles River Laboratories Italia (Calco, Italy). The diet intervention and the processing of the tissue samples are described in the Supporting Information.

### Statistical analysis

ANOVA was performed to test differences in protein expression between control cells and cells treated with exosomes derived from healthy subjects, exosomes derived from NAFLD patients, and modified exosomes, loaded with OA or PA. In all analyses, controls were the reference category. Statistical significance was set at *P ≤ 0.005 and **P ≤ 0.001. The size of the sample used was determined using the Slovin formula. Post-hoc analysis for multiple comparisons was performed by Bonferroni test; a statistical package was used to perform all statistical analyses (Sigma Stat Stat version 3.1). Randomization have been aimed to achieving comparability of groups since is responsible for achieving initially similar groups and in that which is unknown.

## Results

### Characterization of exosomes

Exosomes were isolated from 20 healthy individuals (Healthy-Exo) and 60 NAFLD patients (NAFLD-Exo) to examine the role of exosomes in maintaining liver damage. Freshly isolated exosomes were characterized in terms of size, morphology and surface charge (Fig. S1B, S1D, S1F, S1H, S1L); Western blotting analysis was performed on them to investigate the expression of specific key proteins involved in necroptosis, pyroptosis and inflammation processes, namely RIP-1, GSDMD, and TGF-β1, respectively and the results are reported in the Supporting Information (Fig. S1A, S1A’).

### PA and OA are differently present in exosomes from NALFD patients and healthy subjects

**Fig. 1:**
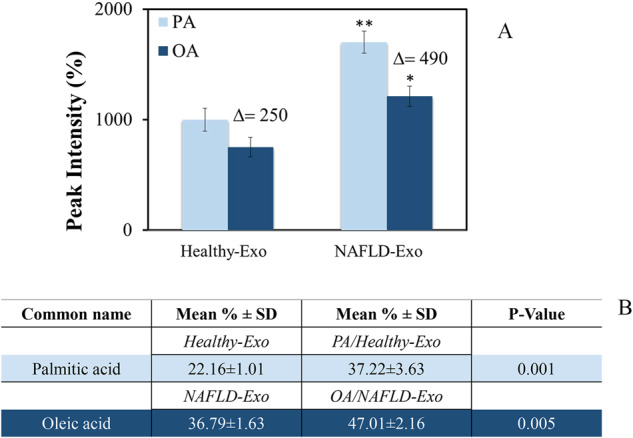
Lipidomic evaluation. **A** Comparison between the peak intensity of PA (light blue bar) and OA (blue bar) obtained by GC-FID chromatography of FA extracted from exosomes of healthy and NAFLD subjects. *P < 0.005 and **P < 0.001. **B** Percentage of PA and OA determined by GC-FID chromatography in exosomes from healthy subjects (Control) and NAFLD patients (NAFLD-Exo), before and after loading with exogenous PA and OA, respectively.

The FA composition of isolated exosomes was investigated using exosome membranes derived from all NAFLD patients and all healthy donors. Overall, in all samples, both the Healthy-Exo and NAFLD-Exo, the PA levels were higher than OA levels (Fig. 1). However, the PA and OA levels were significantly higher (*P* < 0.005 and *P* < 0.001) in the NAFLD-Exo than those in the Healthy-Exo (Fig. 1A). Consistently, the ratio (Δ) between the chromatographic peak intensities of the PA and the OA was also significantly increased (Fig. 1A), yielding *P < 0.005 and **P < 0.001 in NAFLD-Exo as compared to Healthy-Exo samples. In order to assess the toxic effect of the PA/OA imbalance, exogenous OA was added to NAFLD-Exo (OA/NAFLD-Exo) and PA to Healthy-Exo (PA/Healthy-Exo, Fig. 1B and Fig. S1, Supporting Information). The encapsulation efficiency (EE%) of the OA and PA in the exosomes derived from healthy donors and NAFLD subjects was (10.00 ± 3.79)% and (15.06 ± 4.64)%, respectively.

### Effects of exosomes isolated from NAFLD patients and healthy subjects on liver cells

**Fig. 2:**
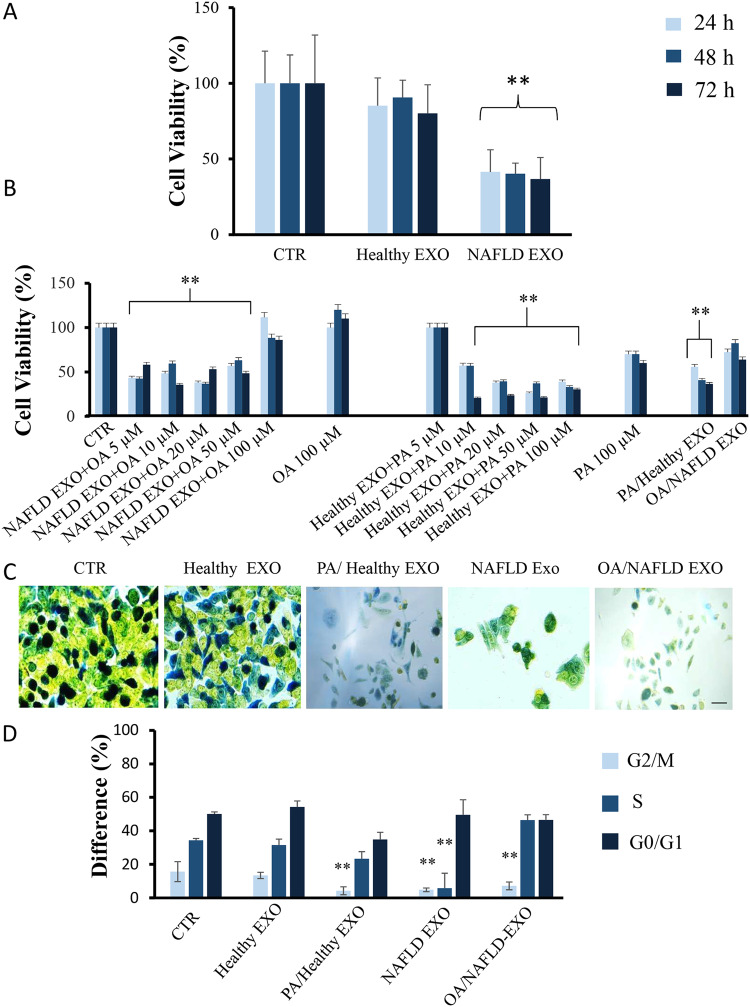
Cell viability evaluation and live cell cycle detection. Cell viability was evaluated by MTS assay, on Hepa-RG cells, after incubation with Healthy-Exo and NAFLD-Exo samples (**A**, **B**), alone or in combination with free PA (fPA) or OA (fOA) at different concentrations (5–100 µM), hybrid exosomes (PA/Healthy-Exo and OA/NAFLD-Exo) and fPA or fOA at 100 μM. Representative bright-field images of live Hepa-RG cells stained with CellClock™ Dye Reagent to monitor the four major cell cycle phases: dark green-blue (G2/M phases), light green (S phase) and Yellow (G0/G1 Phases) (**C**). Cells were treated with exosomes derived from healthy subjects and NAFLD patients, or hybrid exosomes loaded with FA. Scale bar 50 μm. The histogram represents the quantification of differences (%) between the different cell cycle phases (**D**). The exosomes concentration in terms of the total protein content was fixed at 20 µg/µL. The PA and OA concentrations were 10 and 13.5 µM respectively in PA/Healthy-Exo and OA/NAFLD-Exo samples. Negative controls were untreated cells (CTR). Experiments were repeated three times. **P < 0.001.

To investigate the effect of isolated exosomes on liver cells, we challenged Hepa-RG cells with exosomes isolated from 20 healthy donors and 60 NAFLD patients. The cell viability was preserved when 100 μM OA was administered without exosomes, whereas using PA at the same concentration the cell viability was reduced but not in a significant manner. As reported in Fig. 2A, the NAFLD-derived exosomes, but not those derived from healthy donors, significantly (*P* < 0.001) affected cell viability at 24, 48, and 72 h. To better understand the role of the exosome PA/OA imbalance in exerting cell toxicity, experiments were conducted using exogenous free(_f_)-added concentrations (ranging from 5 to 100 µM) of OA and PA to NAFLD and healthy subjects exosomes, respectively. OA added to NAFLD exosomes restored cell viability only at 100 µM, regardless of the incubation time, that ranged from 24 to 72 h. On the other hand, PA_f_ added to healthy donors exosomes significantly affected (*P* < 0.001) cell viability at concentrations ranging from 10 to 100 µM, at every experimental time point (Fig. 2B).

To further demonstrate the effects of the OA/PA ratio, we challenged liver cells with healthy subjects-hybridized exosomes (PA/Healthy-Exo, 13.5 µM PA concentration) and with NAFLD-hybridized exosomes (OA/NAFLD-Exo, 10 µM OA concentration). As shown in Fig. 2A the PA/Healthy-Exo significantly (*P* < 0.001) affected cell viability, while the OA/NAFLD-Exo significantly (*P* < 0.005) restored cell viability, compared to the viability of cells treated with the unmodified NALFD-Exo samples (Fig. 2B).

The Hepa-RG cell cycle activity was analyzed, to investigate the effectiveness of the PA/OA ratio, using the cell clock assay in live cells after treatment for 12 h, with pristine exosomes or FAs loaded hybrid exosomes, as reported in Fig. 2C, D. A significant reduction of color intensity (**=*P* < 0.001) associated to the G2/M phases (dark green/blue cells) and S phase (light green cells) was observed in the cells treated with exosomes isolated from 60 NAFLD patients (NAFLD-Exo), as compared to controls (untreated cells).

Similarly, a significant G2/M phase arrest was induced when Hepa-RG cells were incubated with PA-loaded exosomes isolated from healthy subjects (PA/Healthy-Exo). Exosomes from healthy donors (Healthy-Exo) had no impact on cell cycle activity. However, treatment of cells with OA/ NAFLD-Exo samples induced the arrest of only the S phase. In conclusion, a PA/OA imbalance can be considered responsible for cell viability and arresting the cell cycle.

**Fig. 3:**
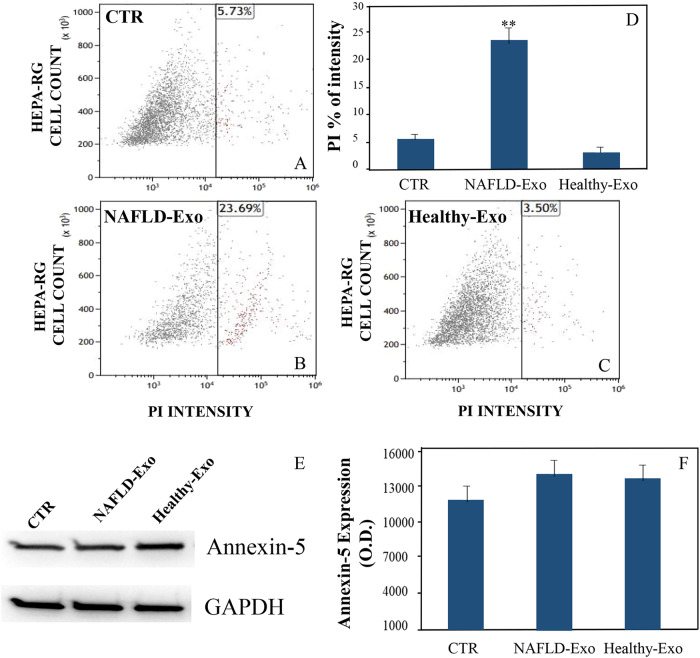
Cell death evaluation by PI and Annexin-5. Cell death was evaluated by PI assay, on Hepa-RG cells, untreated (CTR) (**A**), and after incubation with NAFLD-Exo (**B**) and Healthy-Exo samples (**C**). Experiments were repeated three times and the PI% intensity was reported in (**D**). **P < 0.001. Western blotting assay was performed for Annexin V. Negative controls were untreated cells normalized using GAPDH in the same way as the cells treated with NAFLD-Exo and Healthy-Exo samples (**E**). In the histogram, semi-quantitative evaluation, performed by means of video-densitometry, of the relative expression levels of the Annexin-5 (**F**, average of three experiments). For all the experiments, the exosomes concentration was fixed at 20 µg/µL in terms of total protein content.

To dissect the molecular mechanism involved in liver cell death, we also performed experiments using PI staining and the Annexin-5 assay. A significant increase (**P < 0.001) of cells death, when treated with exosomes derived from patients affected by NAFLD (Fig. 3B vs A), is detected by cytofluorimeter-assisted PI assay, thus confirming data obtained with MTS assay. On the contrary, cells treated with exosomes derived from healthy subjects do not show an increase of cell death (Fig. 3C vs A). To corroborate the results derived from PI, the expression of Annexin-5 was evaluated by western blotting analysis. In Fig. 3E a constancy of expression of this protein can be observed, thus excluding its involvement in cell death, upon 6 h of treatment, as also confirmed by the quantification analysis, indicating that no significant variation occurred (Fig. 3F). To confirm the findings obtained by using the Hepa-RG cell line, we treated primary hepatocytes with exosomes derived from plasma of NAFLD and healthy subjects and investigated early necroptosis by PI and Annexin-5 assays (see Supporting Information, Fig. S2).

**Fig. 4:**
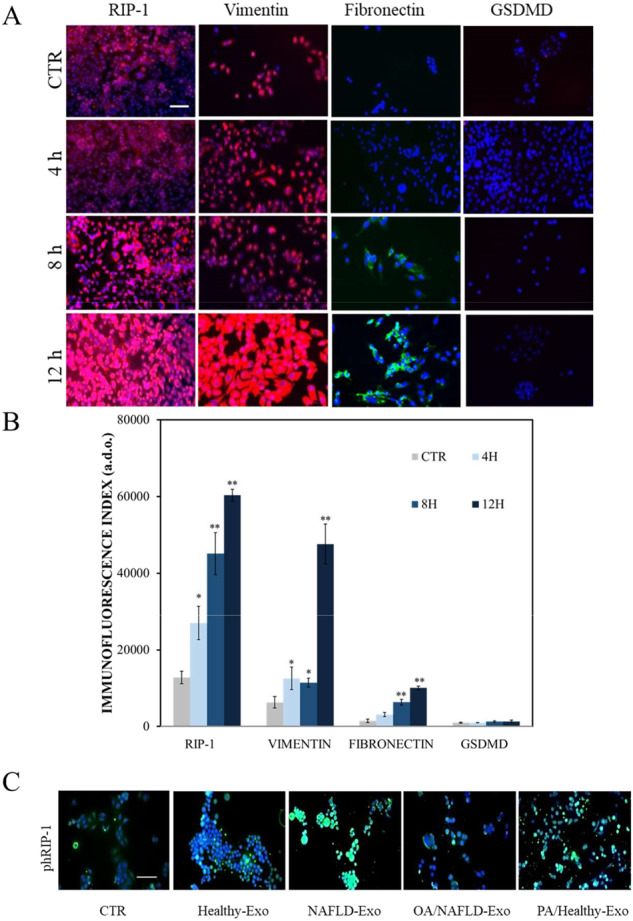
Detection of RIP-1, fibronectin, vimentin, GSDMD and phospho-RIP-1 in Hepa-RG cells by immunofluorescence confocal microscopy. **A** Immunofluorescence imaging and analysis. **B** RIP-1, fibronectin, vimentin and GSDMD in cells incubated for 4, 8, and 12 h with NAFLD-Exo. (**P < 0.001). Blue channel: nuclei; red channel: RIP-1 or vimentin or GSDMD, green channel: fibronectin. **C** Immunofluorescence imaging and phospho-RIP-1 in Hepa-RG cells treated with pristine exosomes (Healthy-Exo and NAFLD-Exo) and hybrid exosomes (PA/Healthy-Exo and OA/NAFLD-Exo) for 12 h. Blue channel: nuclei, green channel: phospho-RIP-1. Experiments were repeated three times. Scale bar 50 μm.

To better understand the liver cell damage induced by a PA/OA imbalance, the expression of specific proteins involved in the initial re-organization of cells fibers, namely vimentin and fibronectin [28, 29] was investigated by confocal microscopy, along with the expression of the RIP-1 and GSDMD proteins, markers for necroptosis and pyroptosis, respectively. Fibronectin was expressed at higher levels in Hepa-RG cells treated with NAFLD-Exo compared to controls, after 8 and 12 h (*P* < 0.001) of treatment, while vimentin was expressed at significantly higher levels after 4, 8 (*p* < 0.005), and 12 h (*p* < 0.001). NAFLD-Exo induced a significantly increased expression of RIP-1 in Hepa-RG cells, after 4 h (*P < 0.005), 8, and 12 h (**P < 0.001). The expression pattern of RIP-1 staining was mainly as small, defined areas or large, diffuse areas at the level of the nucleus and cytoplasm, respectively (Fig. 4A, B). GSDMD was not detected at any of the investigated time points (Fig. 4A, B).

NAFLD-Exo administration to cells resulted in a significantly (*P* < 0.001) increased expression of RIP-1 in the cytoplasm of Hepa-RG cells (Fig. 4A, B), while phospho-RIP-1, was strongly expressed in the nuclei (Fig. 4C). Consistently, weak staining of phospho-RIP-1 was observed in cells treated with NAFLD hybrid exosomes loaded with OA (OA/NAFLD-Exo), whereas intense staining was detected in the nuclei of cells treated with hybrid exosomes from healthy subjects loaded with PA (PA/Healthy-Exo) (Fig. 4C).

**Fig. 5:**
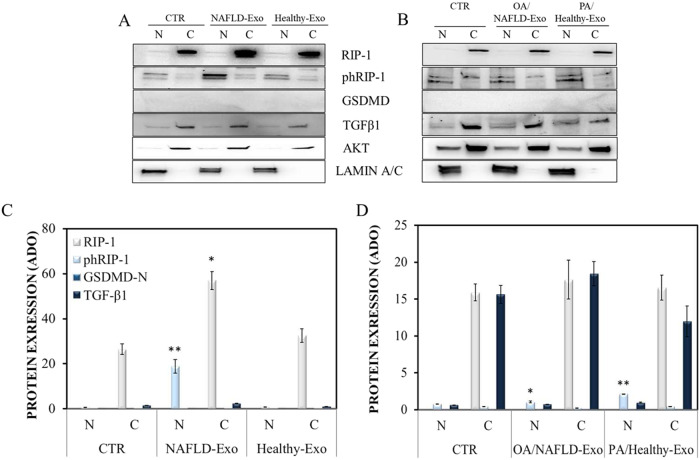
Intracellular expression of RIP-1, phospho-RIP-1, GSDMD, and TGF-β1 in Hepa-RG cells. Representative western blotting of Hepa-RG cells treated with pristine exosomes (**A**) and hybrid exosomes (**B**) for RIP-1, phospho-RIP-1, GSDMD and TGF-β1, normalized using AKT and LAMIN A/C housekeeping proteins for the cytoplasm (C) and nuclei (N), respectively. Semi-quantitative evaluation, performed by means of video-densitometry, of the relative expression levels of RIP-1, phospho-RIP-1, GSDMD, and TGF-β1, normalized using AKT and LAMIN A/C for the cytoplasm, and nuclei, respectively, in the cells treated with exosomes and hybrid exosomes, **C** and **D**, respectively. Negative controls are untreated cells. *P < 0.001 and **P < 0.005. For all the experiments, the exosomes concentration was fixed at 20 µg/µL in terms of total protein content. The PA and OA concentration was 10 and 13.5 µM in PA/Healthy-Exo and OA/NAFLD-Exo samples, respectively.

To confirm the immunofluorescence data, the RIP-1, phospho-RIP-1, GSDMD, and TGF-β1 expression was investigated by western blotting analysis (Fig. 5). RIP-1 was expressed in the cytoplasm but not in the nuclei, whereas phospho-RIP-1 was mostly expressed in the nuclei after treatment with exosomes derived from NAFLD patients, but not from healthy subjects, supporting the hypothesis that ripoptosome complex phosphorylation and nuclear translocation are required for phospho-RIP-1 to exert its function in the necroptotic pathway [14]. Consistently with the results described above, treatment with hybrid exosomes from NAFLD patients loaded with OA decreased phospho-RIP-1 expression in the nuclei, as well as treatment with hybrid exosomes from healthy subjects loaded with PA increased phospho-RIP-1 in the nuclei (Fig. 5).

In conclusion, adding OA or PA to exosomes from NAFLD patients and healthy subjects, respectively, reversed phospho-RIP-1 expression (Fig. 5). Increased expression of phospho-RIP-1 suggests the involvement of the necroptosis rather than pyroptosis pathway in human hepatocyte cell death.

### Exosomes derived from NAFLD patients increase ELOVL6, RIP-1, MLKL, and ROS promoting necroptosis

**Fig. 6:**
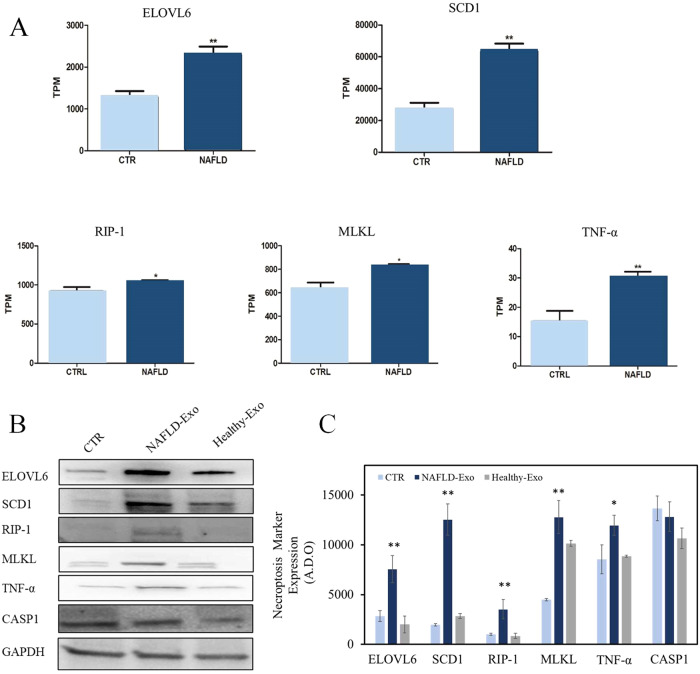
ELOVL6, SCD1, RIP-1, MLKL, TNF-α, and CASP1 mRNA expression in liver tissue of NAFLD patients. mRNA and cellular expression proteins. Data were obtained from RNA sequencing analysis, as downloaded from the Gene Expression Omnibus database (GSE130970), of liver tissue obtained from 72 patients with NAFLD and from 6 controls (CTRL). Mean expression data are expressed as TPM (transcripts per million) (**A**). Representative western blotting analysis of ELOVL6, SCD1, RIP-1, MLKL, TNF-α, CASP1 proteins, and marker protein GAPDH in Hepa-RG cells treated with NAFLD-Exo and Healthy-Exo samples (**B**). Semiquantitative evaluation of proteins expression level in the Hepa-RG cell line treated with NAFLD-Exo and Healthy-Exo samples, by video-densitometry analysis of proteins bands on western blotting. GAPDH protein band was used for the normalization of the targeted protein bands for each sample considered. *P < 0.005 and **P < 0.001 vs healthy subjects or untreated cells (CTR) (**C**).

In order to identify the mediators of the necroptosis process in the NAFLD patients, the gene expression profiles of liver tissue, analyzed in 72 patients with NAFLD and 6 histologically normal subjects (CTRL), were downloaded from the Gene-Expression-Omnibus-database (GSE130970) [30]. ELOVL6, Stearoyl-CoA desaturase-1 (SCD1), RIP-1, MLKL, and Tumor necrosis factor α (TNF-α) mRNA expression were found to be significantly upregulated in NAFLD patients compared to healthy controls (*P* < 0.05) (Fig. 6A). Hepa-RG cells stimulated with exosomes derived from 20 NAFLD patients expressed significantly higher levels (*P < 0.05 or **P < 0.001) of ELOVL6, SCD1, TNF-α, RIP-1, and MLKL, as compared to Hepa-RG cells treated with exosomes derived from healthy subjects. On the contrary, no significant modification of CASP1 expression occurred, suggesting a necroptosis rather than pyroptosis mechanism (Fig. 6B, C). These findings were confirmed by investigating the expression levels of the specific necroptosis markers, namely RIP-1, MLKL, TNF-α, also in primary human hepatocytes (Fig. S3, Supporting Information).

**Fig. 7:**
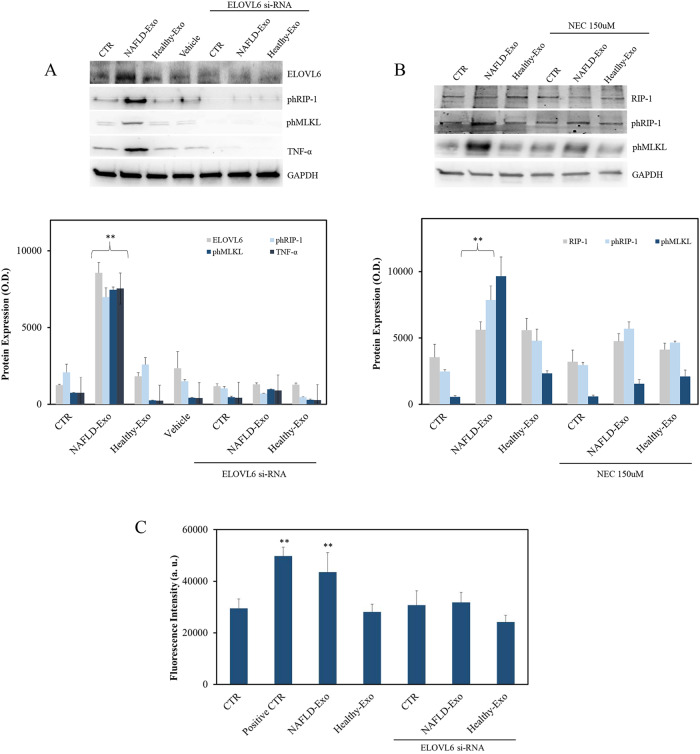
ELOVL6, phospho-RIP-1, and phospho-MLKL expression in Hepa-RG cells. **A** Representative western blotting of ELOVL6, phospho-RIP-1, and phospho-MLKL (upper panel) with the corresponding densitometry analysis of protein level (lower panel) in silenced Hepa-RG cells, after incubation with pristine exosomes only or in combination with transfection complex; 20 μg of total protein extract were loaded. Negative control (CTR) was untreated cells and the vehicle was cells with si‐PORT‐NeoFX transfection agent only; GAPDH was used to normalize the targeted protein for each sample considered. **P < 0.001 vs negative control. **B** Representative western blotting of RIP-1, phospho-RIP-1 and phospho-MLKL (upper panel) with the corresponding densitometry analysis of protein level (lower panel), upon incubation of Hepa-RG cells with exosomes, only or in combination with Necrostatin-1 (NEC); 20 μg of total protein extract were loaded. Negative control (CTR) was untreated cells and GAPDH was used to normalize the targeted protein for each sample considered. **P < 0.001 vs negative control. **C** ROS evaluation by oxidation of 2,7-dichlorophluorescein diacetate (DCFH-DA) upon incubation of Hepa-RG cells with exosomes, only or in combination with transfection complex. Negative control (CTR) was untreated cells, while positive CTR was cells treated with H_2_O_2._ *P < 0.005 vs negative control.

To corroborate previously described results, we performed ELOVL6 RNA silencing experiments, and the expression of ELOVL6, phospho-RIP-1, phospho-MLKL, and TNF-α were investigated by western blotting analysis. In Fig. 7A, the expression of all proteins in Hepa-RG cell line, treated or not with ELOVL6-siRNA, confirmed its determinant role in the activation pathway of phospho-RIP-1. Particularly, for the first time it is possible to observe, in the cells treated with exosomes derived from NAFLD patients, the significantly increased expression (**P < 0.001) of ELOVL6, TNF-α, phospho-RIP-1 and phospho-MLKL, compared to the control and to the cells treated with exosomes derived from the healthy subjects, while in the silenced cells, the signal due to the presence of TNF-α, phospho-RIP-1, and phospho-MLKL was strongly reduced, thus confirming the predominant role of ELOVL6 in the necroptosis pathway activation. Furthermore, inhibition experiments were also carried out with Necrostatin-1, an exclusive inhibitor of the autophosphorylation activity of RIP-1 kinase at different concentrations before the treatment with exosomes derived from healthy and NAFLD affected subjects. As can be seen in Fig. 7B, treatment with Necrostatin-1 before the exosomes addition confirms the fundamental role of NAFLD patients exosomes in RIP-1 phosphorylation and necroptosis activation; what can be observed, in cells treated for 24 h with Necrostatin-1, and then treated with exosomes from healthy subjects and subjects affected by NAFLD for 48 h, is a significant decrease in phospho-RIP-1 (Fig. 7B, **P < 0.001) compared to the expression of this protein in cells treated only with exosomes. Moreover, there was no variation in the cells treated with exosomes from healthy subjects, silenced or not, and in the controls, in the presence or absence of Necrostatin-1, thus indicating that the precise content carried by the exosomes of NAFLD patients is the cause of necroptosis in treated hepatocytes.

Next, we investigated the mechanism of ELOV6 on ph-RIP1 by measuring levels of ROS based on the oxidation of DCFH-DA. Hepa-RG cells treated with exosomes from ten patients with NAFLD produced significantly (*P* > 0.001) higher levels of ROS, 5 h after exosomes administration compared to treatment with Healthy-Exo samples. On the contrary, the increased ROS production was completely inhibited when ELOVL-6 was silenced before exosome treatment (Fig. 7C), suggesting that ELOVL-6 plays a role in ROS production.

### ELOVL6, Phospho-RIP-1, and phospho-MLKL are increased in the liver of mice fed with a saturated fatty acids-enriched diet

**Fig. 8:**
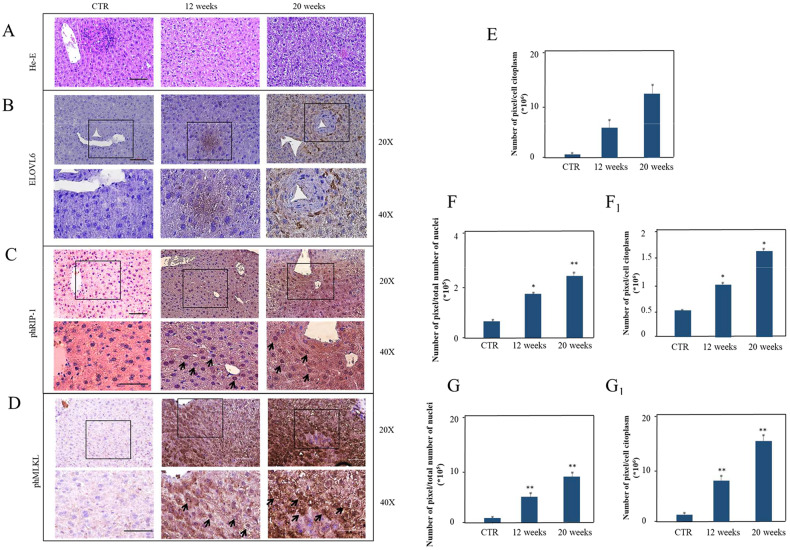
Histology of mice liver tissue. He-E in mice tissues, WT (without treatment) and at 12 and 20 weeks of treatment with a high fat diet (**A**). Immunohistochemical expression of ELOVL6 (**B**), phospho-RIP-1 (**C**) and phospho-MLKL (**D**) in mice without treatment (WT) and fed a high fat diet for 12 weeks and 20 weeks (**B**). Number of pixels per cell cytoplasm or total number of nuclei, mean ± SD obtained for ELOVL6 only for cytoplasm (**E**), phospho-RIP-1 expression in the nuclei (**F**) and cytoplasm (**F**_**1**_). Number of pixels per cell cytoplasm or total number of nuclei, mean ± SD obtained for phospho-MLKL expression in the nuclei (**G**) and cytoplasm (**G**_**1**_). The values observed in the different cellular districts were significantly different by ANOVA test **P < 0.001 and *P < 0.005. Scale bar 50 μm. Black arrows indicate the presence of phospho-RIP-1 or phospho-MLKL in the nuclei.

To verify our findings in an in vivo model, adult mice were fed with a FAs enriched diet and the onset of liver steatosis was histologically investigated. He-E staining confirmed the presence of vacuolar degeneration, larger hepatocytes with a higher nucleus-cytoplasm ratio and a rearrangement of the lobular architecture already after 12 weeks, that became more evident after 20 weeks (Fig. 8A). In the liver of animals fed with a fatty diet, ELOVL6 expression was significantly higher (**P < 0.001) in the cytoplasm after 12 and 20 weeks (Fig. 8B, E) compared to the control. Furthermore, an higher expression of phospho-RIP-1 and phospho- MLKL (*P < 0.005 and **P < 0.001, respectively) in the nuclei after 12 and 20 weeks (Fig. 8C, D), and in the cytoplasm after 12 (*P < 0.005) and 20 weeks (**P < 0.001) for phospho-RIP-1 (Fig. 8F, F_1_) were detected, while the expression of phospho-MLKL increased significantly and early, within 12 weeks (**P < 0.001) in the cytoplasm (Fig. 8G, G_1_). These data suggest that necroptosis also occurs in vivo, being responsible for the liver damage in NAFLD. To corroborate these results, the co-expression of cleaved Caspase-3, Ki-67, or Ki-67 and ph-RIP1 was investigated, reported in Fig. S3 of Supporting Information.

## Discussion

In this study, we demonstrate for the first time that an imbalanced ratio between PA and OA is responsible for liver damage, through the Elongase-6 activation pathway, that ultimately leads to necroptosis. This conclusion is based on the following evidence: (i) the PA/OA ratio in exosomes isolated from NAFLD patients is imbalanced; (ii) exosomes isolated from NAFLD patients induced a reduction of cell viability and proliferation in Hepa-RG cell line; (iii) loading of exogenous OA into exosomes isolated from NAFLD patients restores cell viability in Hepa-RG cell line, while the load of PA in exosomes isolated from healthy subjects shows cytotoxic effects even more than the PA administered as free fatty acid at the same concentration as that encapsulated in exosomes; (iv) hybrid exosomes from healthy subjects affect cell viability and proliferation when administeredto Hepa-RG, whereas those from NAFLD patients do not; (v) the absence of Elongase-6, as well as the use of Necrostatin-1, prevents RIP-1 phosphorylation, thus restoring the cell viability, upon treatment with exosomes derived from NAFLD patients; (vi) CASP1-GSDMD axis pyroptosis-mediated, is not involved in the cells death derived from the PA/OA exosome imbalance.

The PA/OA ratio, evaluated using the lipidomic assay on the exosomes derived from NAFLD patients, is consistent with the evidence reported on the erythrocytes membranes, found to be richer in PA that can induce an early activation of apoptosis, than OA that induces a decrease of intracellular lipid and inflammation markers levels as compared to those from healthy subjects [31–34]. Furthermore, in NAFLD subjects, a higher PA concentration has been linked to a lower hepatocyte viability [35], as well as to changes in the cell cycle, thus reflecting a reduction in the regeneration rate which is, ultimately, counteracted by cellular hyperplasia events [36]. In experimental models of hepatic steatosis, PA generates liver damage, while OA results less harmful, capable of preventing/attenuating PA-induced toxicity in in vitro models [10, 37]. Moreover, OA improves PA-mediated hepatocellular lipotoxicity by inducing pyroptosis in different types of cells, such as β-cells, hepatocyte and muscle cells [30, 38, 39]. In accordance with the reported literature, our study shows the effects of PA, carried by circulating exosomes derived from NAFLD patients, on cell survival and proliferation. Furthermore, it has been shown that artificially PA-enriched exosomes, derived from healthy subjects, attain the toxicity of those derived from NAFLD patients, while the free fatty acid administered to the cells even in high concentrations (100 μM), causes a reduced mortality compared to the encapsulated PA at the same concentration, indicating an increased toxicity when conveyed in the exosomes. On the contrary, cells treated with OA-loaded exosomes from NAFLD patients preserve the normal cellular metabolic activities, cellular viability and cell cycle, without notable side effects, and the same dose of free OA administered to the cells preserves the cell viability.

According to the literature, while the plasma membranes remain intact and the DNA is fragmented, during the apoptosis process, early stages of necroptosis lead to plasma membrane pore formation, evidenced by PI staining and Annexin-5 unaltered levels [40, 41]. Based on previous knowledge, we employed PI-assisted flow cytometry to confirm the occurrence of an early necroptosis cell death event after 6 h post exosomes administration, considering that a complete exosome absorption takes up to 4 h [42]. The fast PI absorption observed and the absence of changes in Annexin-5 levels in cells treated with NAFLD- or healthy subjects-derived exosomes allowed us to discern the different cell death pathways. In addition, we observed no changes, upon treatment, in the caspase-1 level, a hallmark in the apoptosis pathway [43].

Recent literature describes beneficial effects of a diet enriched with nontoxic unsaturated FAs, including monounsaturated and polyunsaturated fatty acids (MUFA and PUFA), to prevent PA induced lipotoxicity [44], cardiovascular risk, NAFLD, and diabetes [45]. Moreover, the presence of an overexpression of genes related to fibrosis (such as vimentin and fibronectin) has been revealed in hepatic stellate cells treated with exosomes secreted from PA-treated hepatocytes [46]. The genetic profile of liver tissue derived from patients with NAFLD, reported in the Gene Expression Omnibus database, shows an overexpression of lipogenic enzymes, such as ELOVL6, SCD1, both usually absent in normal hepatocytes, and associated with a decreased hepatic steatosis and adiposity [47], as well as proteins like TNF-α, MLKL and RIP-1, which have been linked to an augmented uptake of PA [31]. RIP-1 regulates inflammation and cell death by interacting with receptor-interacting serine/threonine protein kinases 3 (RIP-3). The phosphorylation of RIP-1 plays a critical role in programmed necrosis signaling, as shown in mass spectroscopy studies [48, 49]. Several papers reported that the auto-phosphorylation of RIP1 is regulated by the increase of ROS [21]. TNF-α has been recently reported to be a potent mediator of ROS production [50], thus leading to damage of hepatocytes in patients with Non-alcoholic Steatohepatitis, where an increase of EVOLV-6 has also been described [51]. Following these reports, we describe here the molecular mechanism by which the exosome PA/OA imbalance, referred as toxic, mediates liver damage, employing in vitro and ex vivo approaches. We ruled out that a chronic exposure to those exosomes switches the hepatocytes toward a NAFLD-like genotype and phenotype, by inducing ELOVL6 and SCD1 expression, which, in turn, activated TNF-α, RIP-1, and MLKL mediated necroptosis [52]. We also demonstrated a consistent activation of necroptotic activity in cells treated with hybrid exosomes, derived from healthy subjects and loaded with PA, as a result of the increased RIP-1 phosphorylation within the nuclei of hepatic cells, that was completely attenuated in the presence of Necrostatin-1, a specific necroptosis inhibitor, constantly used in literature. Finally, our data also revealed no change in the expression of CASP1, GSDMD and TGF-β1 proteins, under any of the conditions studied, ruling out a contribution of the inflammasome in this cell death pathway, mediated by the ELOVL6-TNF-α-ROS-RIP-1-MLKL axis, and explaining the absence of inflammation in NAFLD.

## Conclusion

Our results highlight for the first time that exosomes derived from NAFLD patients could be responsible for the progression of the disease, as primary vectors of a PA/OA ratio imbalance. ELOVL6, an enzyme that converts PA to OA, is a key player in the activation of RIP-1 and, consequently, responsible for the necroptosis pathway activation by the ELOVL6-TNF-α-ROS-RIP-1-MLKL axis. Furthermore, if properly enriched with OA, exosomes could allow a metabolic re-balancing and a reduction of liver steatosis. Indeed, the use of hybrid exosomes might be seen as one of the initial steps in the approach to a more personalized therapy for an orphan disease like NAFLD.

### Supplementary information

Supporting Information
