# Supplementary material for: The oleic/palmitic acid imbalance in exosomes isolated from NAFLD patients induces necroptosis of liver cells via the elongase-6/RIP-1 pathway
Source: Cell Death Dis. 2023 Sep 26;14(9):635. doi: 10.1038/s41419-023-06161-9 (PMC10522611; doi:10.1038/s41419-023-06161-9)
Supplement: Supplementary file 1 — Supporting Information [file 41419_2023_6161_MOESM1_ESM.docx]

**Supporting Information**

The Oleic/Palmitic acid imbalance in exosomes isolated from NAFLD patients induces necroptosis of liver cells via Elongase-6/Rip-1 pathway

The exosomal Oleic/Palmitic acid imbalance induces necroptosis in liver cells

Maria Principia Scavo^1*#^, Roberto Negro^1*#^, Valentina Arrè^1*^, Nicoletta Depalo^2^, Livianna Carrieri^1^, Federica Rizzi^2^, Rita Mastrogiacomo^2,3^, Grazia Serino^4^, Maria Notarnicola^5^, Valentina De Nunzio^5^, Tamara Lippolis^5^, Pasqua Letizia Pesole^6^, Sergio Coletta^6^, Raffaele Armentano^6^, Maria Lucia Curri^2,3^, Gianluigi Giannelli^7^

1-Personalized Medicine Laboratory, National Institute of Gastroenterology “S. de Bellis” , IRCCS Research Hospital, Via Turi 27, Castellana Grotte, 70013 Bari, Italy; [maria.scavo@irccsdebellis.it](mailto:maria.scavo@irccsdebellis.it); [roberto.negro@irccsdebellis.it](mailto:roberto.negro@irccsdebellis.it); [valentina.arre@irccsdebellis.it](mailto:valentina.arre@irccsdebellis.it); [livianna.carrieri@irccsdebellis.it](mailto:livianna.carrieri@irccsdebellis.it).

2-Institute for Chemical-Physical Processes (IPCF)-CNR SS Bari, Via Orabona 4, 70125 Bari, Italy; [n.depalo@ba.ipcf.cnr.it](mailto:n.depalo@ba.ipcf.cnr.it); [f.rizzi@ba.ipcf.cnr.it](mailto:f.rizzi@ba.ipcf.cnr.it)n.depalo@ba.ipcf.cnr.it;f.rizzi@ba.ipcf.cnr.it.

3-Dipartimento di Chimica, Università degli Studi di Bari Aldo Moro, Via Orabona 4, 70125 Bari, Italy; rita.mastrogiacomo@uniba.it; [marialucia.curri@uniba.it](mailto:marialucia.curri@uniba.it).

4-Experimental Immunopathology Laboratory, National Institute of Gastroenterology “S. de Bellis” IRCCS Research Hospital, Via Turi 27, Castellana Grotte, 70013 Bari, Italy; [grazia.serino@irccsdebellis.it](mailto:grazia.serino@irccsdebellis.it).

5-Laboratory of Nutritional Biochemistry, National Institute of Gastroenterology “S. de Bellis”, IRCCS Research Hospital, Via Turi 27, Castellana Grotte, 70013 Bari, Italy; [maria.notarnicola@irccsdebellis.it](mailto:maria.notarnicola@irccsdebellis.it); [valentina.denunzio@irccsdebellis.it](mailto:valentina.denunzio@irccsdebellis.it).

6-Department of Pathology, National Institute of Gastroenterology “S. de Bellis” IRCCS Research Hospital, Via Turi 27, Castellana Grotte, 70013 Bari, Italy; [letizia.pesole@irccsdebellis.it](mailto:letizia.pesole@irccsdebellis.it); [raffaele.armentano@irccsdebellis.it](mailto:raffaele.armentano@irccsdebellis.it); sergio.coletta@irccsdebellis.it.

7-Scientific Direction, National Institute of Gastroenterology “S. de Bellis” IRCCS Research Hospital, Via Turi 27, Castellana Grotte, 70013 Bari, Italy; [gianluigi.giannelli@irccsdebellis.it](mailto:phosphor#.giannelli@irccsdebellis.it).

* Correspondence: maria.scavo@irccsdebellis.it; roberto.negro@irccsdebellis.it; Tel.: +39-080-4994697;

# M.P.S., R.N. and V. A. have equally contributed to this study.

**Author Agreement**: All authors have seen and approved the final version of the manuscript being submitted.

**Materials and Methods**

*Patients*

This trial, conducted at the National Institute of Digestive Diseases' Laboratory of Epidemiology and Biostatistics, IRCCS "S. de Bellis", Castellana Grotte, Italy, enrolled 60 participants with NAFLD with severe steatosis and without diabetes, as well as 20 healthy subjects. The participants had been invited to take part by their General Practitioners, or identified during the NutriEp recruitment or follow-up procedures. The trial lasted from March 2015 to December 2016. Patients were sequentially enrolled, and the trial was registered at www.clinicaltrials.gov (registration number CT02347696). The study was carried out in compliance with the Helsinki Declaration and was authorized by the Ethics Committee (Prot. n. 10/CE/De Bellis, 3 February 2015).

*Animals*

20 Wild type male mice were randomly assigned to four groups, each of five animals (2 control groups for a total of 10 animals ), one group (5 animals) fed for 12 and another one fed for 20 weeks (5 animals) as follows: 2 control (ST) groups were fed a purified AIN93M standard diet (12.5% protein, 12% soybean oil, 3% cellulose fiber) for 12 (5 mice) and 20 weeks (5 mice); 2 (5 mice for each group) treated groups were fed a high-fat low-fiber diet (5K20: 18.5% protein, 10.5% fat, of which 1.5% saturated fatty acids, 4.5% soluble and insoluble fibers, 7% ashes, and 60% non-nitrogenous chemicals (Mucedola Srl, Settimo Milanese, Italy)). After 12 or 20 weeks of dietary treatment, the animals were sacrificed by cervical dislocation and samples of whole liver tissue were removed, collected, paraffin-embedded, and processed for histochemistry.

*Cell Culture*

Human Hepa-RG cell line (Thermo Fisher Scientific, Waltham, Massachusetts, United States cod. HPRGC10 ) was cultured using a hepatocyte bullet kit medium [500 mL of HBM Medium, 10 mL BSA-FAF (fatty acid free, transferrin 0.5 mL, insulin recombinant human 0.5 mL, rhEGF in buffer BSA saline solution 0.5 mL, hydrocortisone 21-hemisuccinate 0.5 mL, ascorbic acid 0.5 mL, GA-1000 gentamicin sulfate amphotericin B 0.5 mL), Lonza cod 185389] added with 10% FBS, depleted of exosomes (Gibco cod. A2720801). Primary hepatocyte cells were cultured as a secondary cell line used to verified our findings derived from Hepa-RG cells. The cultured cell lines was tested for mycoplasma contamination using MycoFluor™ Mycoplasma Detection Kit (Thermo Fisher Scientific, Waltham, Massachusetts, United States, cod M7006). Briefly, human Hepatocyte (Genpur, vial LOHUCPG) were plated for the first time using the human hepatocyte thawing medium (Lonza cod MCHT50), and cultured with HBM (Lonza). When the cells reached the confluence, they were used for the PI and Annexin-5 experiments and for Western blotting analysis to confirm the necroptosis events induced by exosomal PA/OA imbalance.

*Fatty acids gas chromatography quantification*

In a BPX 70 capillary column, SGE Analytical Science, P/N SGE054623, 60 m × 0.25 mm ID – BPX70 0.25 μm (SGE Europe Ltd., Milton Keynes, UK), separation of the FAMEs was carried out. Hydrogen was used as carrier gas, at 3.0 mL/min in constant flow mode. The temperature of the injector and the FID detector was maintained at 250 °C. The initial oven temperature was 40 °C, then increased to 170 °C at 10 °C for 5 minutes, then to 200 °C at 4 °C for another 5 minutes and finally to 240 °C at 10 °C, maintained for 5 minutes. Quantification of the FAMEs was performed using a mixture of standards (Supelco 37 Component FAME Mix, Sigma-Aldrich, Milan, Italy). The encapsulation efficiency (EE%) of the FA loaded in the exosomes was evaluated by GC. Briefly, the stock solutions of both FAs used for the loading of exosomes at a concentration of 500 μM, and the supernatants recovered during the preparation of FA loaded exosomes, containing only FAs not encapsulated in exosomes, were prepared as a methyl esters solution by adding toluene and BF_3_•MeOH (FAME). After incubation, the samples were treated with aqueous NaCl solution and toluene and finally analyzed by GC. The content of FA into the exosomes was quantified by GC, with same method above described.

*Cells Proliferation Assay.*

Hepa-RG cells were treated with exosomes from plasma derived from NAFLD patients and from healthy subjects. Briefly, cells were seeded into 96-well plates at a density of 2 × 10^3^ cells/well. After 24 h, the cells were treated with the exosomes derived from all subjects (both NAFLD patients and healthy controls), at a concentration, in terms of total protein content of the exosomes, equal to 20 µg/µL for 24, 48 and 72 hours, in the absence and presence of one of the two FAs, PA or OA, at different concentrations (1 to 100 μM). For the *in vitro* study on the viability of the Hepa-RG cells treated with FA-loaded exosomes, the protein content concentration was kept to 20 µg/µL while the concentrations of PA and OA were equal to 10 and 13.5 µM, respectively. After cell incubation, cells were treated with the MTS tetrazolium compound (CellTiter 96® AQueous One Solution Cell Proliferation Assay, Promega cod G3582) for three additional hours and the absorbance was measured at a wavelength of 490 nm using a Perkin Elmer Victor Plate Reader (Mechelen, Belgium).

*Propidium iodide (PI) fluorescence and Annexin-5 cell death assay*

After 6 hours of exosomes administration, untreated and treated cells (with Healthy-Exo and NALFD-Exo samples) were collected, including dead floating cells in the medium, washed twice in PBS 1X and re-suspended in 2 μg/mL PI (ImmunoChemistry TECHNOLOGIES cod 638). The percentage of cells which took up PI was measured by flow cytometry (Beckman Coulter-Navios). Furthermore, the cells were treated in the same way for the Annexin-5 assay, to verify if the early cell death was due to apoptosis and not to necroptosis. Briefly, after treating the cells as described above, they were collected, and the total proteins were extracted, dosed with Bradford assay, and used in quantities of 20 µg/µL for Western blotting, according to the protocols described above. The antibodies used are rabbit anti-Annex-5 (1:500 Cell Signaling Technology cod 8555) and mouse anti-GAPDH (1:1000 Abcam cod ab8245) for normalization.

*Immunofluorescence for imaging of stress fibers and markers of necroptosis and pyroptosis*

Immunofluorescence analysis was performed on the Hepa-RG cell line, seeded into sterile chamber slides at a density of 1 × 10^4^ cells/well. After 24 hours, the cells were treated with exosomes from plasma obtained from NAFLD patients (NAFLD-Exo) and healthy subjects (Healthy-Exo), at a total protein content concentration of 20 µg/µL, once daily for 3 days. Every day, a slide chamber for Hepa-RG cells treated with NAFLD-Exo and Healthy-Exo, or the hybrid exosomes loaded with OA or PA (PA/NAFLD-Exo and OA/Healthy-Exo), respectively were fixed with cold 96° ethanol prior to immunofluorescence analysis, performed following the same protocol reported in a previous study [26]. Briefly, after the incubation with primary antibodies, namely Anti-RIP1 (1:400 Invitrogen cod PA5-20811), anti-phospho-RIP1(1:300 Thermo Fisher cod PA5-105640 ), cleaved anti-Gasdermin D (1:400 Cell Signaling, cod 36425 ), Ki-67 (Dako cod M7240), cleaved caspase-3 (1:300 Cell Signaling Technology cod 9661), anti-Vimentin (1:200 Cell Signaling Technology cod 5741), and anti-Fibronectin (1:200 Thermo Fisher cod MA5-11981), treated cells were incubated with a specific green fluorescent conjugated secondary IgG Alexa 488 (Invitrogen cod A32731 ) or red fluorescent conjugated secondary IgG Alexa 555 (Invitrogen cod A32732 ) for 1 h and mounted using Prolong Gold Antifade reagent containing DAPI (Invitrogen cod P-36931 ). Images were acquired with a Nikon Eclipse Ti2 fluorescence microscope and analyzed by using the interactive software installed on the machine for the pixel count/area (NIS Elements), considering the same area surface for all acquisitions. Images were acquired by exciting with Kr-Ar and Ar lasers fitted at 20 ×magnification. The fluorescence intensity was quantified by using an exposure time of 500 ms per acquisition for all the investigated samples.

*Protein Extraction and Quantification from Nuclei and Cytoplasm derived from cell lines*

Western blotting analysis was performed on the total protein content extracted from HEPA-RG cells treated with exosomes or hybrid exosomes, loaded with FA derived from healthy subjects and NAFLD patients with severe steatosis. Untreated cells were used as control. From the whole cells treated with pristine or hybrid exosomes, for the mechanisms study the proteins were extracted and homogenized using 1 × radio immunoprecipitation buffer (RIPA, Cell Signaling Technology, Danvers, MA, USA cod 9806) containing protease inhibitor (Amresco, Solon, OH, USA cod M221). While the nuclear and cytoplasmic extraction and separation were performed with a Ne-PER nuclear and cytoplasmic extraction reagent kit (Thermo scientific cod 78835) using fresh cells. Briefly, 5 × 10^6^ cells were transferred into a 1.5 mL microcentrifuge tube and centrifuged at 500 × g for 2-3 minutes. The supernatant was discarded, and the pellet was dried. Then, ice-cold CER I was added to the cell pellet, and vortexed to fully resuspend the cell pellet. After incubation for 15 minutes on ice, the ice-cold CERII reagent was added to the tube and vortexed for another 5 seconds, followed by an incubation on ice for 1 minute. After centrifugation for 5 minutes at 15000 × g, the supernatant, being the cytoplasmic extract, was immediately transferred to a clean pre-chilled tube. The insoluble pellet was suspended in ice-cold NER solution, treated with a vortex cycle for 15 seconds, and kept on ice for 10 seconds, for a total processing time of 40 minutes. The suspension obtained was centrifuged at 15000 x g for 10 minutes and the supernatant, containing the nuclear extract fraction, was transferred to a clean and pre-chilled tube. The method used for protein extraction, total proteins amount quantification and immunoblotting, was reported in Scavo M. P. et al. [26]. Anti-RIP1 (1:400 Invitrogen cod PA5-20811 ), anti-phospho-RIP1(1:300 Thermo Fisher cod PA5-105640), anti-TGF-β1 and cleaved anti-Gasdermin D (1:1000 and 1:400 Cell Signaling, respectively cod 3711 and cod 39754), anti-AKT and anti-lamin (used both at 1:400 Cell Signaling cod 9272 and cod 2032), anti-ELOVL6 (1:500 abcam, cod ab69857), anti-SCD1 (1:500 Invitrogen cod MA5-27542 ), anti-Caspase-1 (1:1000 Cell Signaling cod 2225), anti-GAPDH (1:1000 Invitrogen cod 437000), and TNF-α (1:500 Invitrogen cod MA5-23720) were used as primary antibodies and the Western blotting membranes were incubated with each of them overnight. Then, the membranes were treated with the corresponding HRP-conjugated secondary antibodies against to mouse or rabbit (1:1000 Santa Cruz, Santa Cruz, CA, USA respectively cod sc-2357 and sc-2005), following the previously reported protocol [26]. The chemiluminescence signals from proteins were imaged after incubation using an enhanced chemiluminescence kit (Bio-Rad, Hercules, CA, USA) and analyzed using Chemidoc XRS + software (Bio-Rad, Hercules, CA, USA). Each experiment was repeated three times.

*Silencing of ELOVL6*

The adherent cells were trypsinized and diluted in normal growth medium for 1 hour before transfection. siPOR-NeoFX transfection agent (5 µL) was diluted in Opti-MEM-I (100 µL) and incubated for 10 minutes at room temperature for each well. Similarly, silencer select FDZ10-siRNA was diluted in Opti-MEM-I medium at room temperature to a final concentration of 5 nM. The transfection complexes were formed by mixing SiPORT-NeoFX transfection agent and ELOVL6-siRNA. The mixture was incubated for 10 minutes at room temperature, then overlayed to the cells. Untreated cells were used as negative controls, while cells treated only with diluted solution of siPOR-NeoFX transfection agent were analyzed as vehicle control. After 48 hours of incubation, the silenced cells were treated with exosomes derived from 10 healthy or 10 NAFLD subjects for 72 hours and the proteins expression was investigated by performing western blotting analysis by using anti ELOVL6, phospho-RIP-1, phospho-MLKL (Thermo Fisher PA5-105678) and GAPDH antibodies previously described.

*Inhibition of RIP-1 in HEPA-RG cell line by using Necrostatin-1*

Necrostatin-1 solution at different final concentration from 50 to 200 μM was used. Briefly HEPA-RG cells were seeded into sterile 6-well culture plates at a density of 2 × 105 cells/well and treated with or not with Necrostatin-1 at different concentration described before. After 24 hours the cells were treated with Healthy-Exo and NAFLD-Exo samples and incubated for 48 hours. For each concentration an untreated control and a control treated with only Necrostatin-1 were analyzed. After proteins extraction procedures the western blotting was performed and the hybridization of membrane was conducted for RIP-1 and phospho-RIP-1 for all samples, with the protocol previously described.

*Immunohistochemistry on mice liver tissue*

The procedures related to animal use were communicated to the Italian Ministry of Health and approved. Wild type male mice were randomly divided into 4 groups of 5 animals each, and fed as follows: 2 control (ST) groups received a purified AIN93M standard diet (12.5% protein, 12% soybean oil, 3% cellulose fiber), for 12 (5 mice) and 20 weeks (5 mice); the 2 treated groups received a high-fat low-fiber diet (5K20: 18.5% protein, 10.5% fat, 4.5% soluble and insoluble fibers, 7% ashes and 60% non-nitrogenous compounds; Mucedola Srl, Settimo Milanese, Italy) for 12 (5 mice)) and 20 weeks (5 mice). All diets were isocaloric and supplied as pellets (Mucedola Srl, Settimo Milanese, Italy) and mouse body weights were recorded weekly. After 12 or 20 weeks of dietary treatment, all animals were euthanized by cervical dislocation and samples of whole liver tissue were removed, collected, paraffin-embedded and cut into 4 μm thick slices, Consecutive sections were obtained from each slice and stained with hematoxylin–eosin (He-E, Sigma-Aldrich) or processed for Immunohistochemistry. Anti-phospho-RIP-1 (Thermo Fisher Scientific), anti-phospho-MLKL (Cell signaling) and anti-ELOVL6 were used, according to the procedures described below. After antigen retrieval by microwave irradiation in citric buffer at pH 6.0, slides were incubated with primary antibodies overnight at 4 °C. The antibodies were diluted 1:50 with phosphate buffered saline (PBS, Gibco). The antibody reaction was detected with a polymer-based visualization kit (EnVision, Dako A/S, Glostrup, Denmark), according to the manufacturer’s instructions, using 3,3-diaminobenzidine-tetrahydrochloride (DAB, Vector laboratories) as the chromogen, and Harris hematoxylin (Sigma-Aldrich) for nuclear contrast. Images were acquired with a Nikon Eclipse Ti2 fluorescence microscope in brightfield and analyzed by using the software installed on the microscope (NIS Elements), for the pixel count/area, considering the same area surface for all acquisitions or the same number of nuclei. The images were acquired at 20 × and 40 × magnification.

**Results**

***Characterization of Exosomes***

Western blotting analysis (Figure S1A) was performed on the freshly isolated exosomes to investigate the expression of specific key proteins involved in necroptosis, pyroptosis and
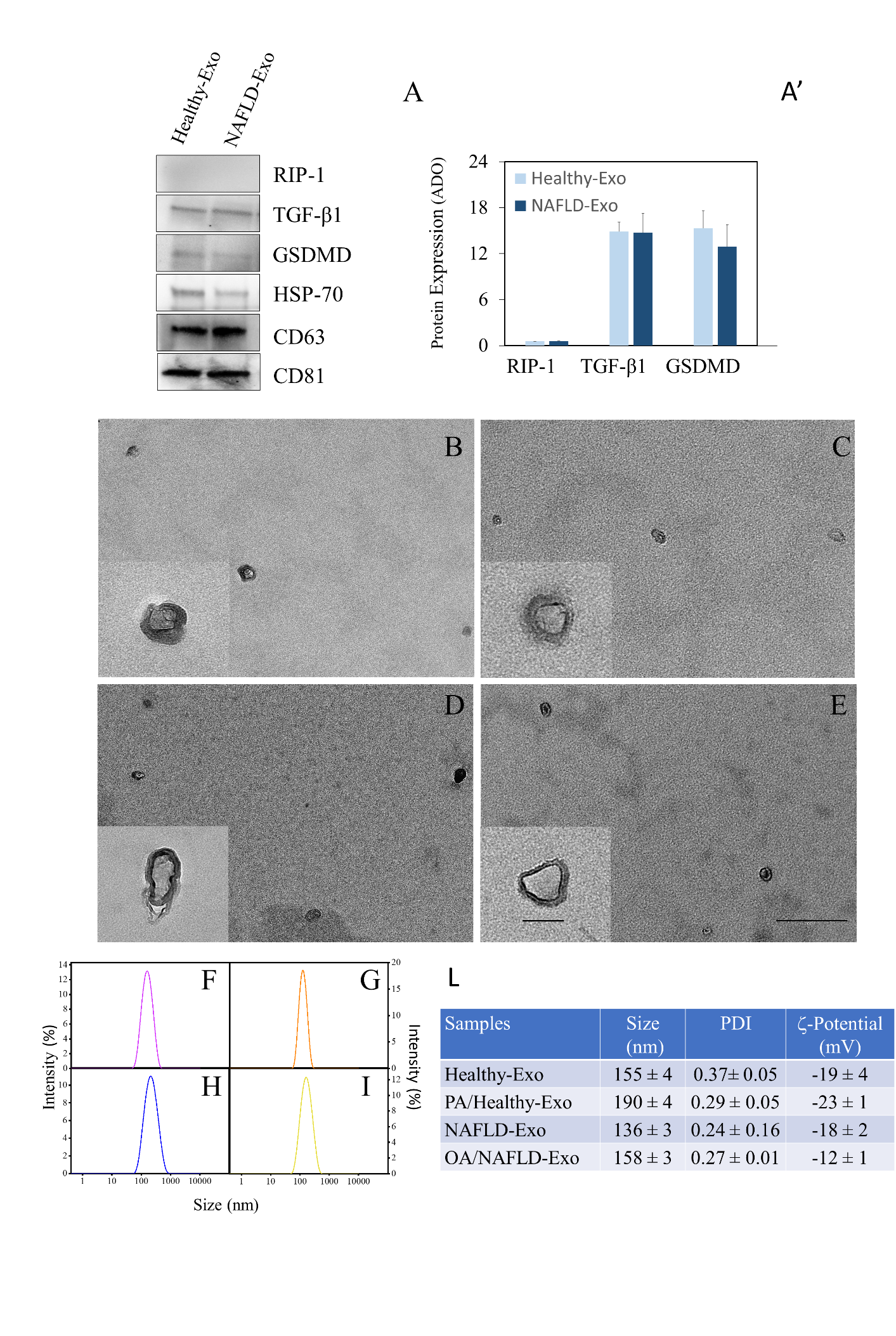


***Figure S1****:* ***Exosome characterization****. (A) Representative western blot analysis of RIP-1, TGF-β1, GSDMD,* *HSP-70, CD63 and CD81 in Healthy-Exo and NAFLD-Exo samples. HSP-70, CD63 and CD81 are three housekeeping proteins for exosomes (A’) Semi-quantitative evaluation, by video-densitometry analysis, of the relative expression levels of RIP-1, TGF-β1 and GSDMD in protein content extracted from exosomes (ado = arbitrary optical density). RIP-1, TGF-β1 and GSDMD were normalized to the housekeeping protein CD81. Representative TEM micrographs and size distribution by DLS of* *Healthy-Exo (B, F) and NAFLD-Exo (C, G), and their corresponding hybrid FA loaded exosomes, namely PA/Healthy-Exo (D, H) and OA/NAFLD-Exo (E, I). (Scale bar 200 nm, Inset: 50 nm). Intensity-average hydrodynamic diameter and corresponding polydispersity index (PDI) obtained by DLS analysis (F-I, L) and ζ-potential value (L) of the exosomes, before and after loading with FAs (mean ± SD, n = 3).*

inflammation processes, namely RIP-1, GSDMD and TGF-β1, respectively. Both healthy donors (Healthy-Exo) and NAFLD patient (NAFLD-Exo) exosomes lack RIP-1 expression, while all samples showed similar levels of GSDMD and TGF-β1 (Figure S1A'). TEM micrographs of Healthy-Exo and NAFLD-Exo patients show spherical nanovesicles featuring a bilayer structure (Figure S1B and S1C). All exosomes isolated presented an average hydrodynamic diameter of about 155 nm and 136 nm, respectively, according to the DLS analysis (Figures S1F, S1L). ζ-potential measurements highlighted the presence of a negative charge on exosomes surfaces, consistent with the expected presence of phospholipid-based cell membranes. An original procedure was used to obtain hybrid exosomes loaded with FAs. In particular, Control were loaded with PA (PA/Healthy-Exo), while the NAFLD-Exo with OA (OA/NAFLD-Exo). TEM investigation proved that no change occurred in their morphology after the loading process with FAs (Figure S1D and S1E). Although their size remained smaller than 200 nm, a slight increase of the diameter occurred in both the exosome preparations, that reached 190 nm for the FAs loaded hybrid exosomes prepared from healthy donors and 158 nm those from NAFLD patients (Figure S1H, S1I and S1L). All of the hybrid exosomes were found to preserve a negative charge (Figure S1L).

***Induction of necroptosis due to the imbalance of PA/OA on primary hepatocyte cells.***

To further confirm the mechanism underpinning liver cells death, we used the same experimental approaches in primary hepatocytes, namely as PI staining, Annexin-5 detection and western blotting analysis for the evaluation of ELOVL6, SCD1, RIP-1, MLKL, TNF-α and CASP1 level.

- *Propidium iodide (PI) fluorescence and Annexin-5 cell death assay on primary hepatocyte cells*

A significant increase in the PI uptake (**P<0.001) from primary hepatocyte was observed upon treatment with NAFLD-Exo (Figure S2B vs S2A). On contrary, cells treated with Heathy-Exo did not show an increase of cell death (Figure S2C vs S2A). Figure S2D summarizes triplicate experiments. To corroborate the results obtained from the PI staining, the expression of Annexin-5 was evaluated by western blot in primary cells treated with exosomes derived from NAFLD and healthy subjects. We didn’t observe changes in the expression level of Annexin-5 among different treatments, which led us to exclude its involvement in cell death upon 6 hours of treatment (Figure S2E and S2F).”


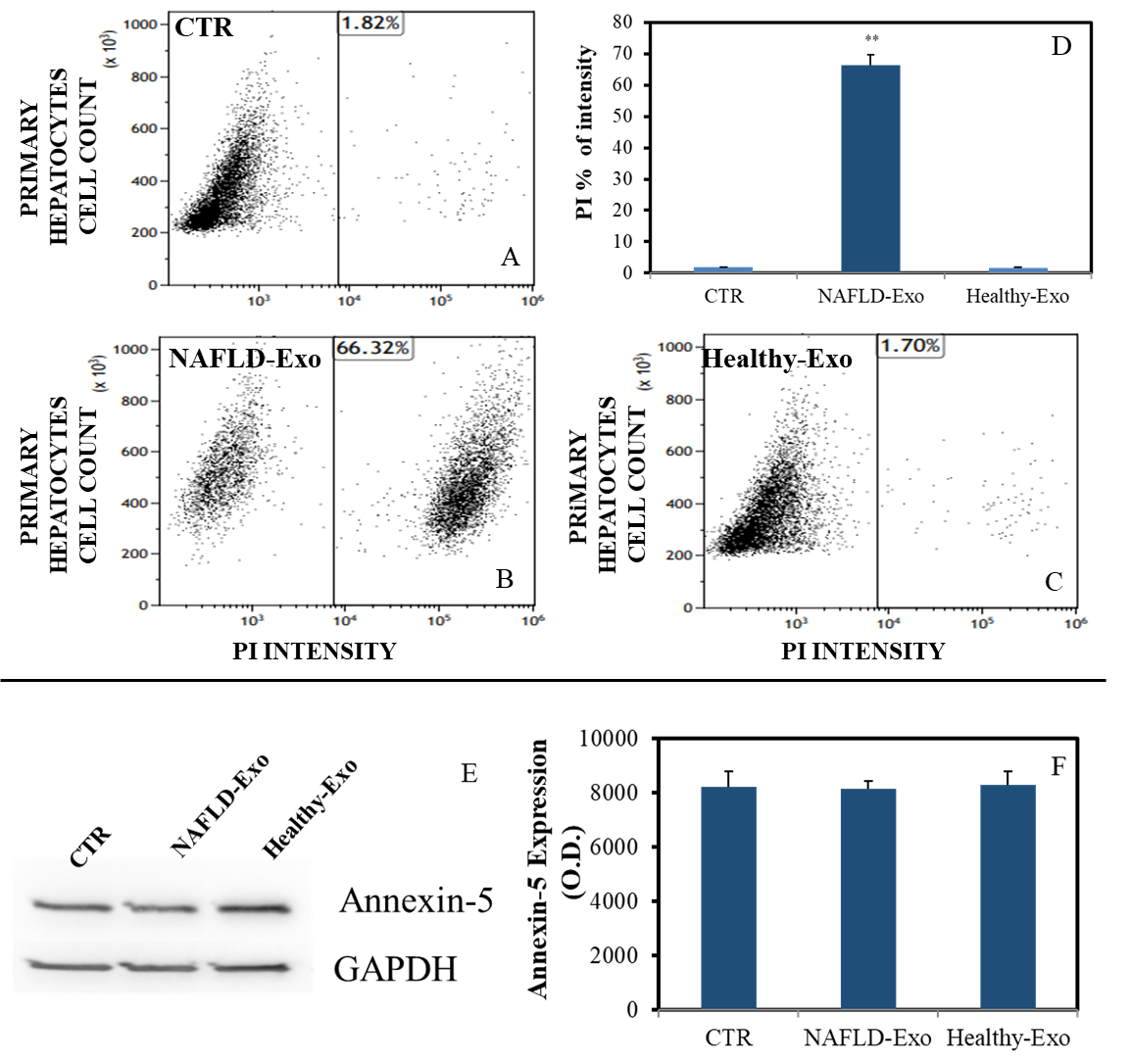


**Figure S2.** ***Primary hepatocyte cell death evaluation by PI and Annexin-5****. Cell death was evaluated by PI assay, on primary hepatocyte cells, untreated (CTR) (A), and after incubation with NAFLD-Exo (B) and Healthy-Exo samples (C). Experiments were repeated three times and the PI % intensity was reported in section D. (**) P<0.001. The exosomes total protein content concentration was fixed at 20 µg/µL and Western blotting assay was performed for Annexin-5. Negative controls are represented by the untreated cells normalized using GAPDH same to the other samples as cells treated with exosomes derived from NAFLD and healthy subjects (E). In the histogram semi-quantitative evaluation, of the relative expression levels of the Annexin V was reported in section F (average of three experiments). For all the experiments, the exosomes concentration was fixed at 20 µg/µL in terms of total protein content.*

- *Exosomes derived from NAFLD patients increase ELOVL6, RIP-1, MLKL and promoting necroptosis in primary hepatocyte cells.*

Primary hepatocytes stimulated with NAFLD-Exo samples, showed a significant increase (*P<0.05 or **P<0.001) of ELOVL6, SCD1, TNF-α, RIP-1 and MLKL expression, as compared to the cells treated with Healthy-Exo. In contrast, the expression of caspase-1 (CASP1) remained unchanged, suggesting a necroptosis pathway rather than pyroptosis mechanism (Figure S3).

*
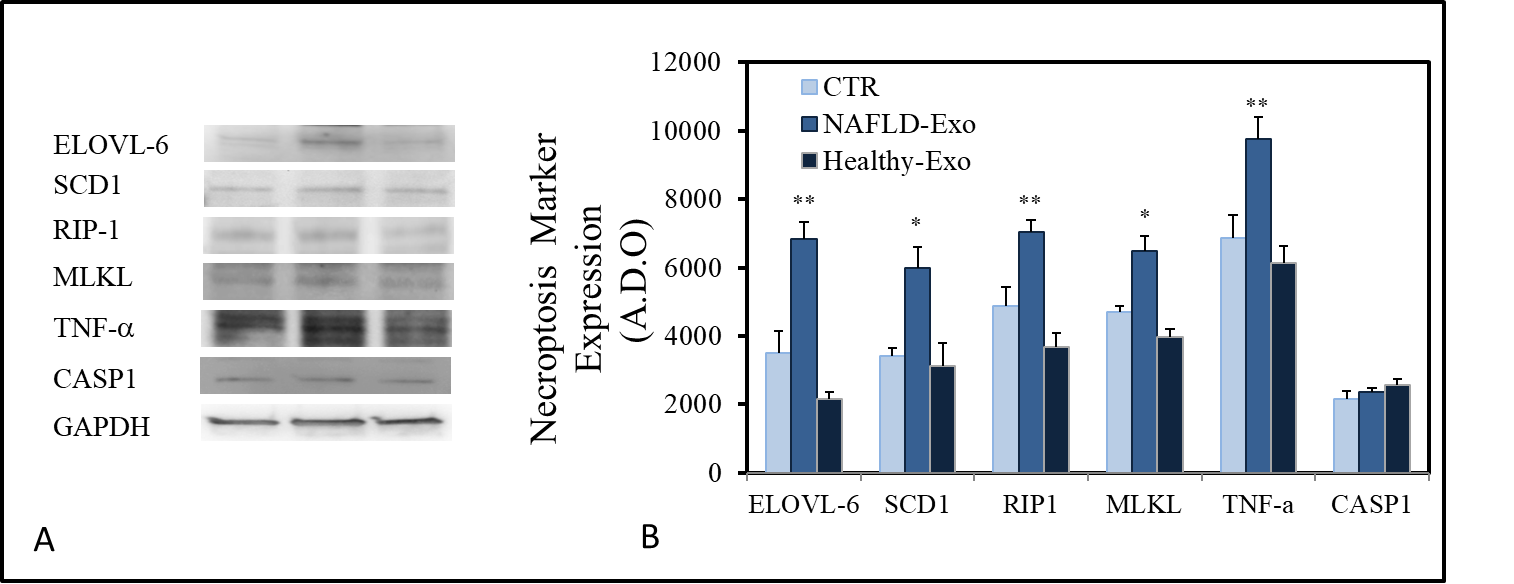
*

**Figure S3**: ***ELOVL6, SCD1, RIP-1, MLKL, TNF-****α* ***and CASP1 expression in primary hepatocyte cells treated with exosomes derived from NAFLD patients and healthy subjects****. Representative western blotting analysis of ELOVL6, SCD1, RIP-1, MLKL, TNF-α, CASP1 proteins and marker protein GAPDH in hepatocyte primary cells treated with exosomes derived from 10 NAFLD patients and healthy donors. Semi-quantitative evaluation of proteins expression level in the hepatocyte primary cells treated with exosomes derived from NAFLD patients and healthy donors, by video-densitometry analysis of proteins bands on western blotting. GAPDH protein band was used for the normalization of the targeted protein bands for each sample considered. (*) P<0.005 and (**) P<0.001 vs healthy subjects or untreated cells (CTR).*

***Immunofluorescence for cell cycle markers and cell death.***

After treatment of adult mice with a FAs enriched diet mice, the immunofluorescence analysis was performed to investigate on co-localization of specific cell cycle markers, namely Ki-67 and cleaved Caspase-3 or Ki-76 and phRIP-1 were performed. Control group (CTR) was represented by mice that received a purified AIN93M standard diet (Figure 4S). There is no significant difference in the expression of active caspase-3 between liver tissues derived from WT mice (CTR) and steatotic liver tissues derived from mice feed with high fat diet (Figure S4A). Moreover, the fluorescence derived from Ki-67 marker in CTR and steatotic samples is very low. Our results add an important aspect to what has been reported in the literature on the prolonged exposure FA-mediated cell death and liver degeneration. Petra Hirsova et al. reported a modest increase of the cleaved caspase-3 expression in liver tissue alongside a compensatory increase of Ki-67 in mouse model of NASH (https://doi.org/10.1038/s41419-020-2283-9). Here we show earlier events in the hepatic degeneration process, in mice treated with high fat diet that, at the time of sacrifice, developed NAFLD rather than NASH.

In the Figure S4B, the presence of Ki-67 is still low and confined to the cytoplasmic part of the cells, while as corroboration of the principal mechanism in steatotic tissue, the ph-RIP1 was expressed in cytoplasmic and in the nuclear compartment in the liver of the mice fed with high-fat low-fiber diet, coherent with the results obtained by the single IHC analysis (Figure 8 of the manuscript).


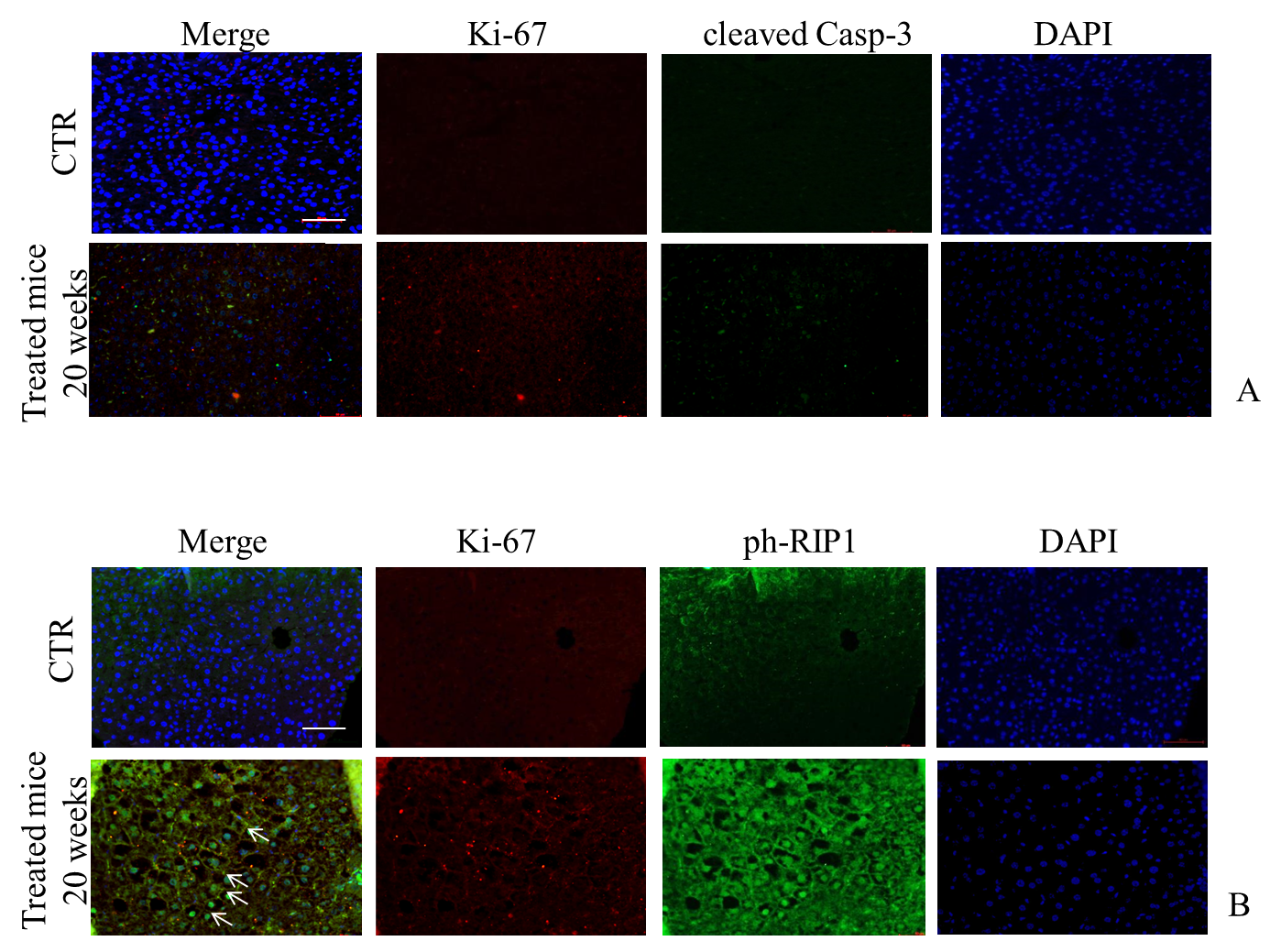


**Figure S4. *Immunofluorescence for Ki67, cleaved caspase-3 and ph-Rip1, in liver tissue from mice treated with high fat diet for 20 weeks.*** *Blue channel: nuclei; red channel: Ki-67 green channel: cleaved caspase-3 (A) or ph-RIP1(B). Experiments were repeated three times. Scale bar 50 μm and the 20X was used as magnification.*

**Original western Blotting**

*Figure S1 RIP1*


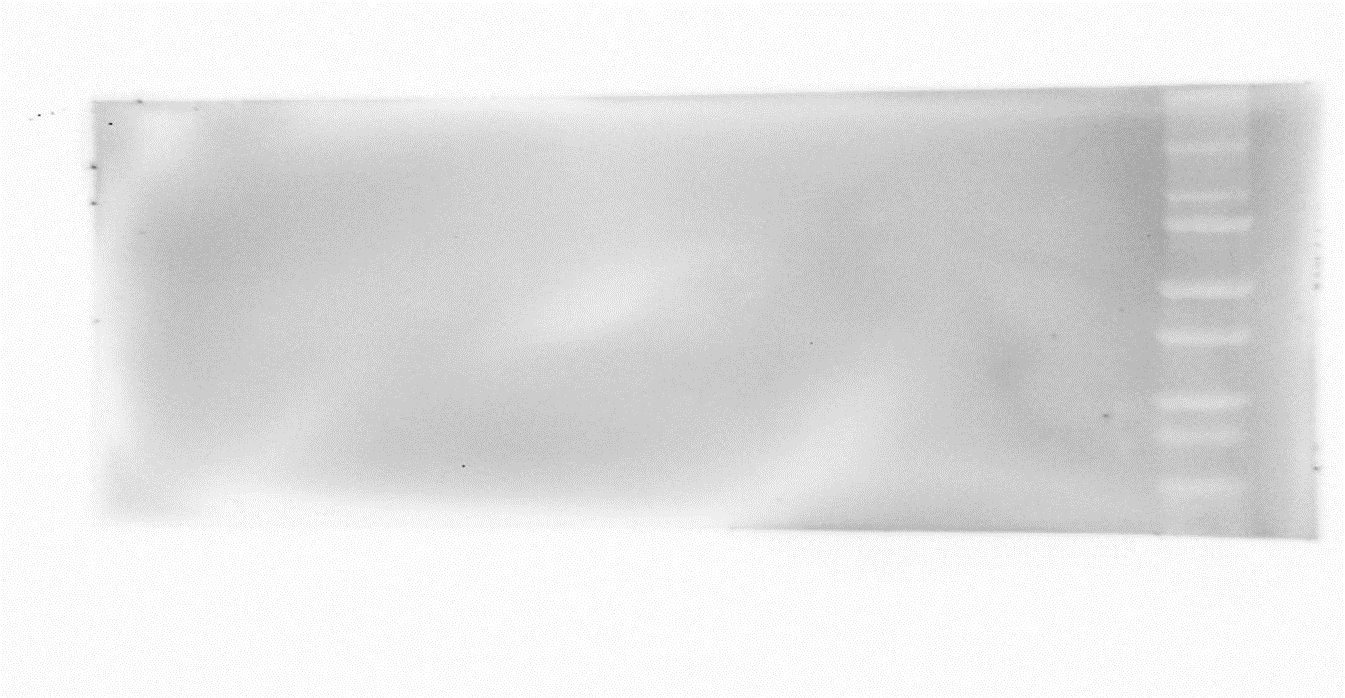


*Figure S1 TGF-b*


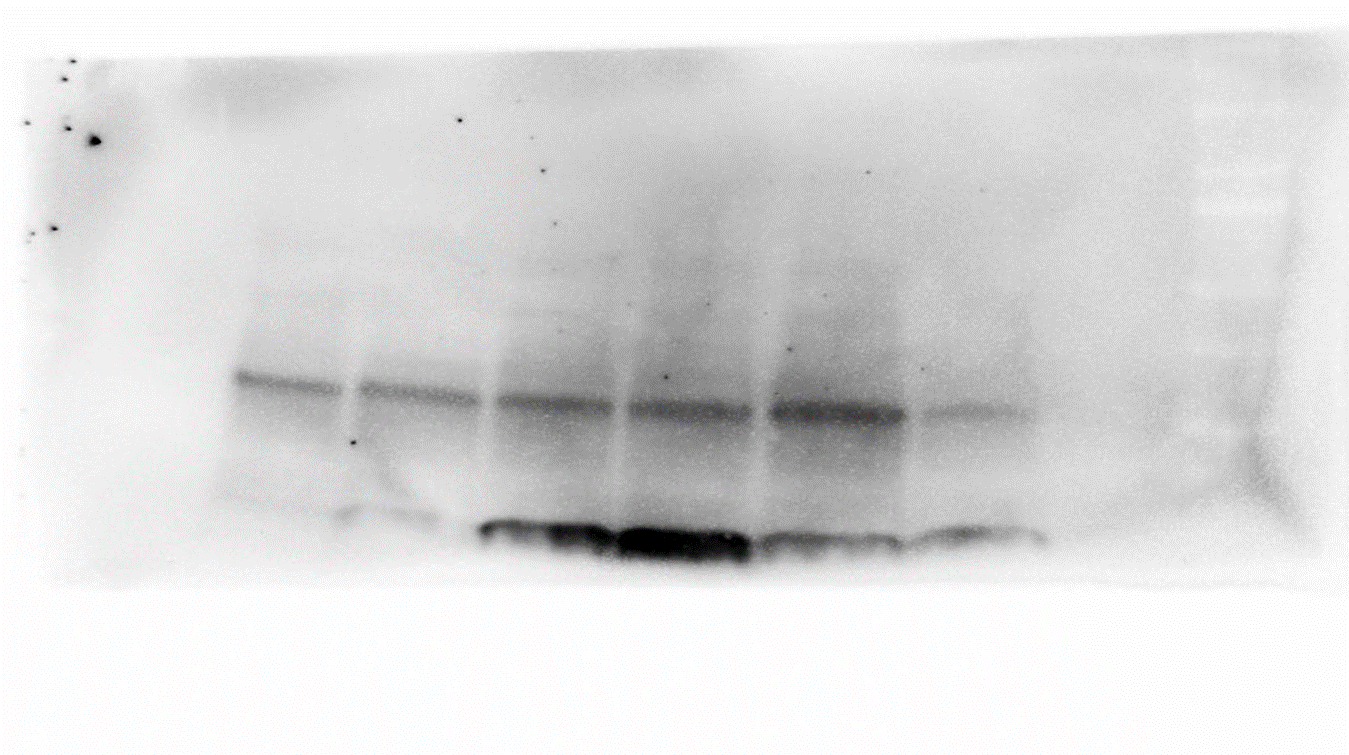


*Figure S1 GSDMD*


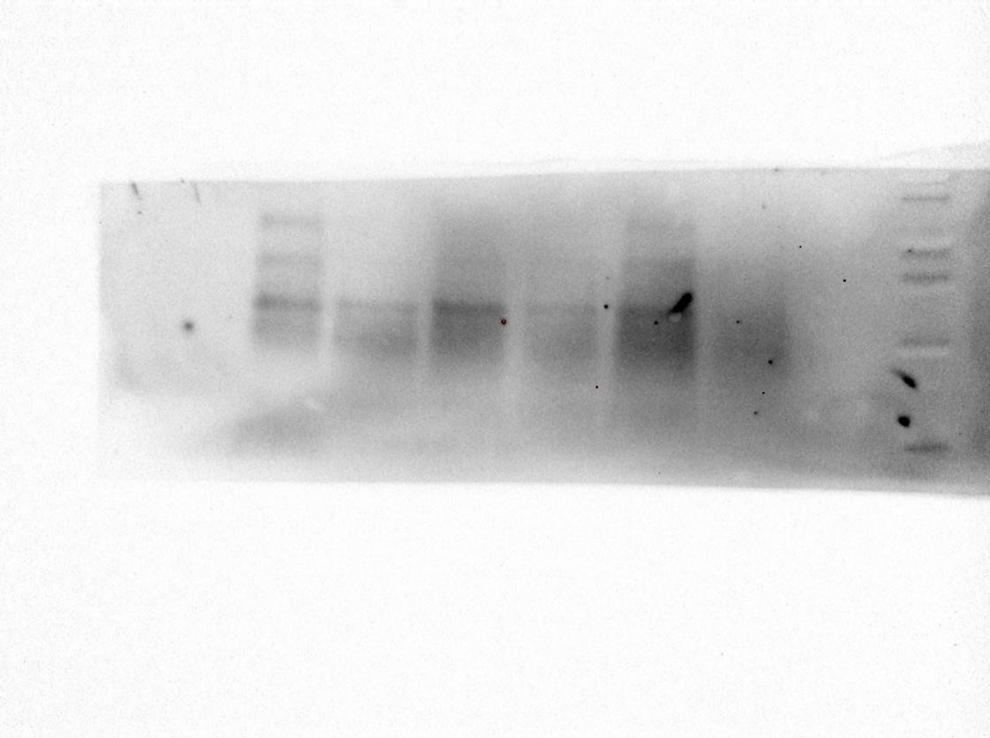


*Figure S1 HSP-70*


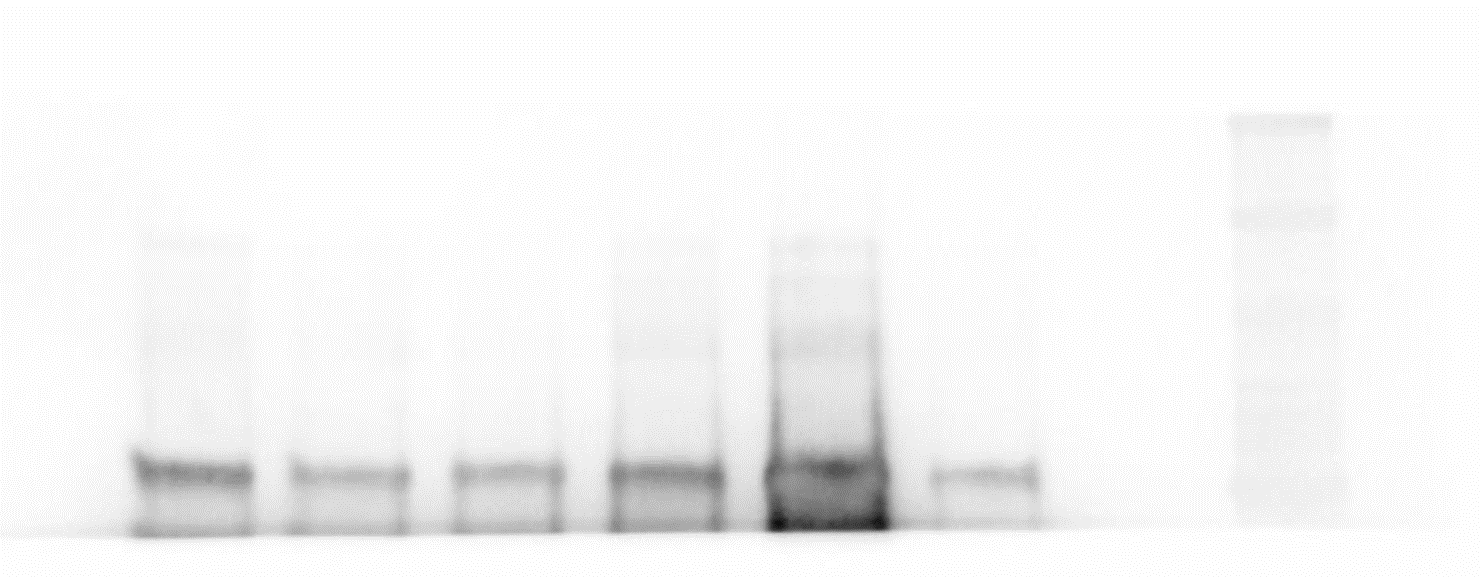


*Figure S1 CD-63*


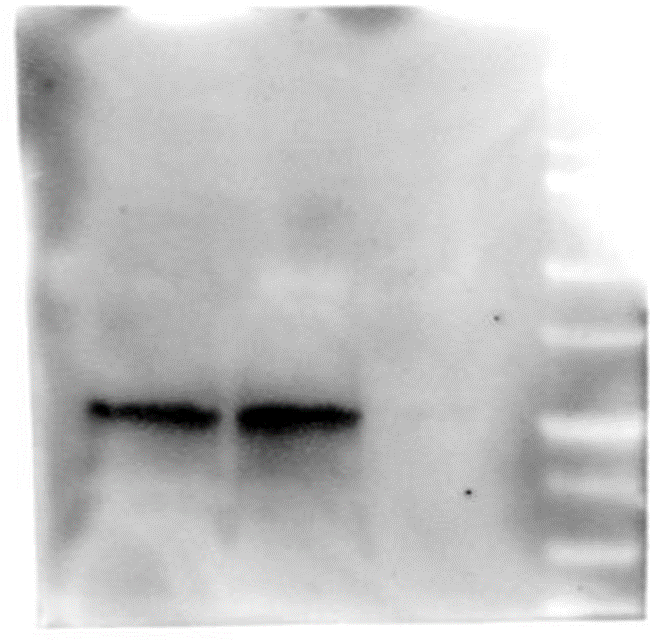


*Figure S1 CD-81*


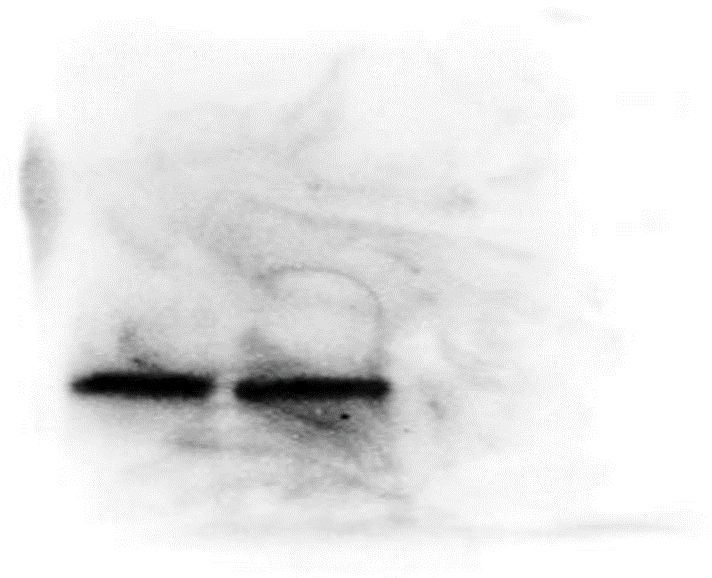


*Figure 3 Annexin-5*


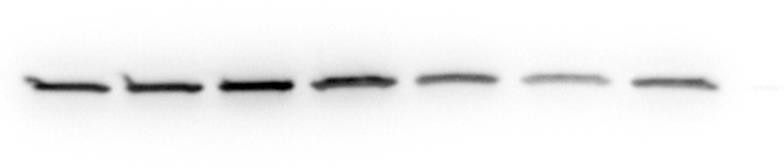


*Figure 3 GAPDH*


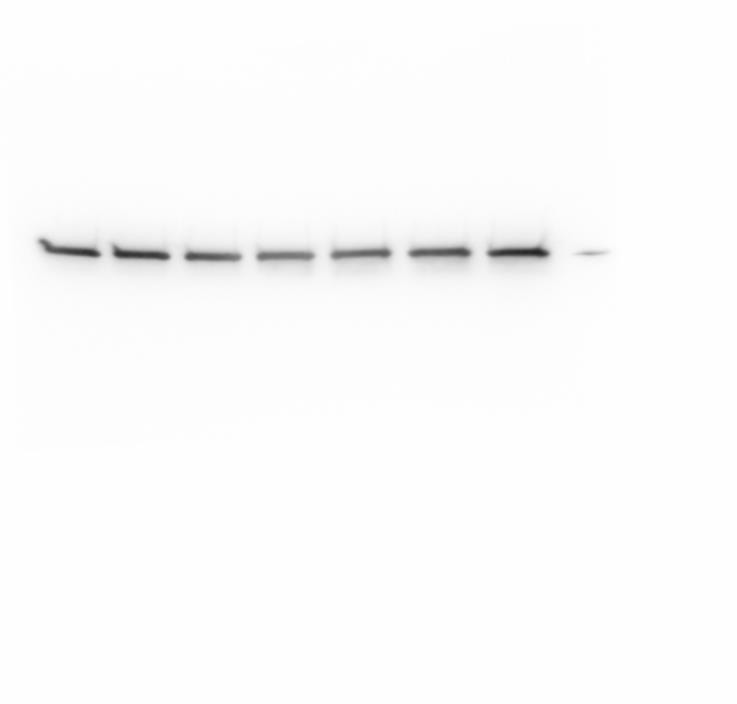


*Figure 5 AKT*


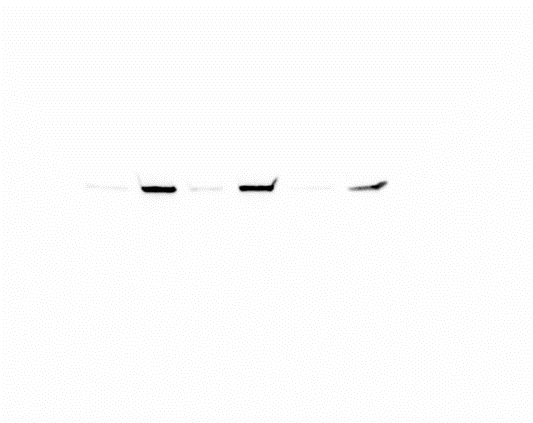


*Figure 5* GSDMD


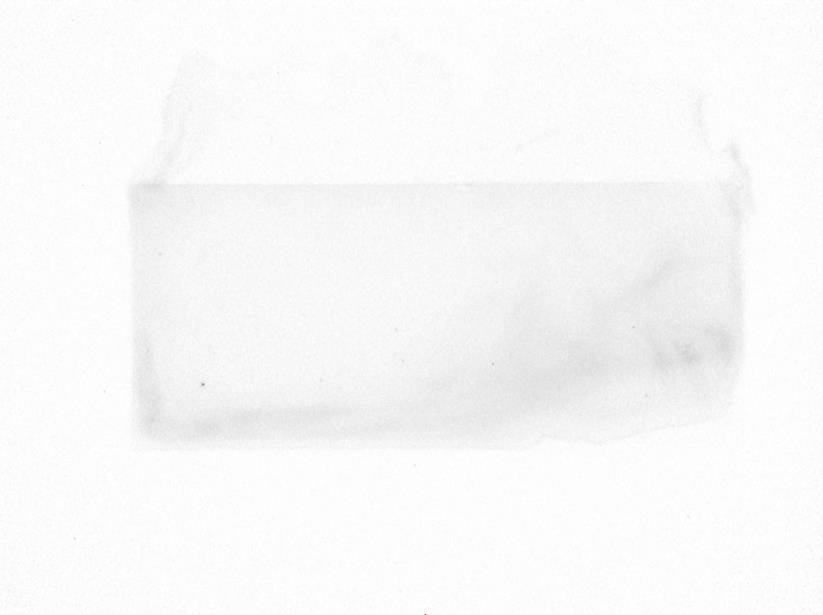


*Figure 5 Lamin*


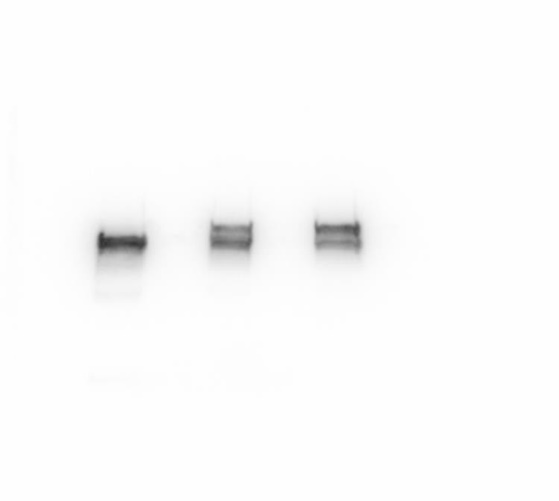

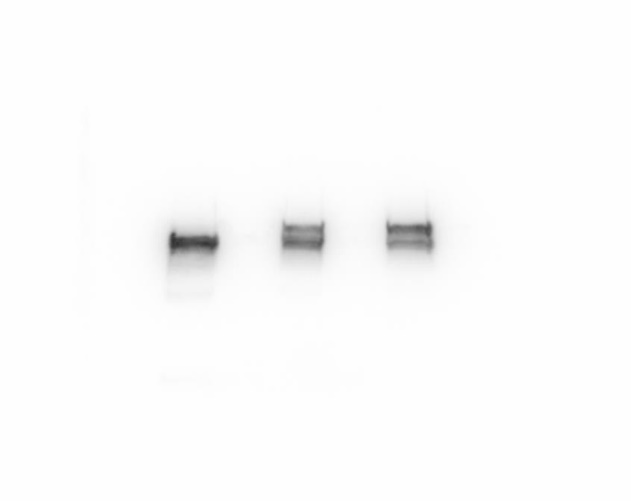


*Figure 5 Rip 1*


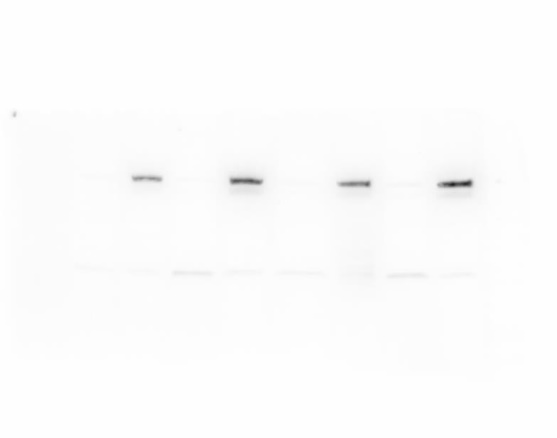


*Figure 5 TGF-b-b*

*
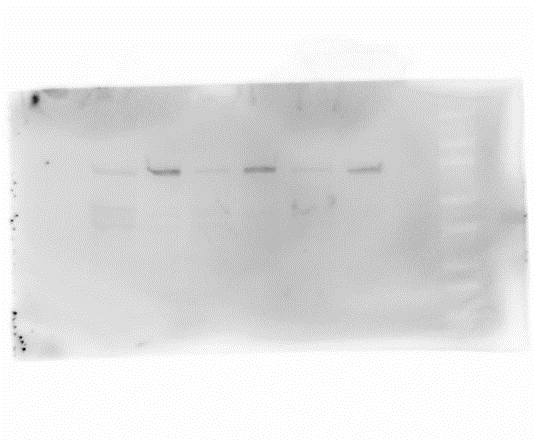
*

*Figure 5 AKT*

*
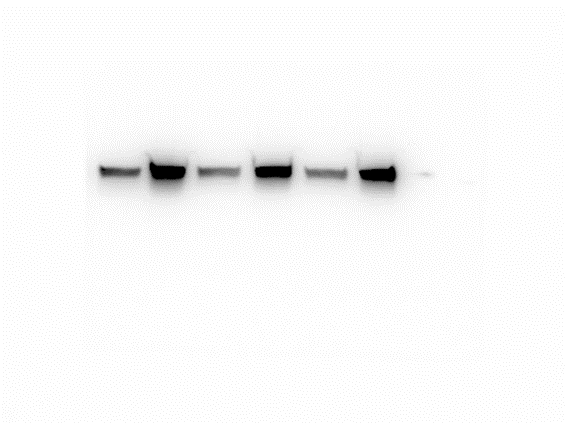
*

*Figure* *5 GSDM*

*
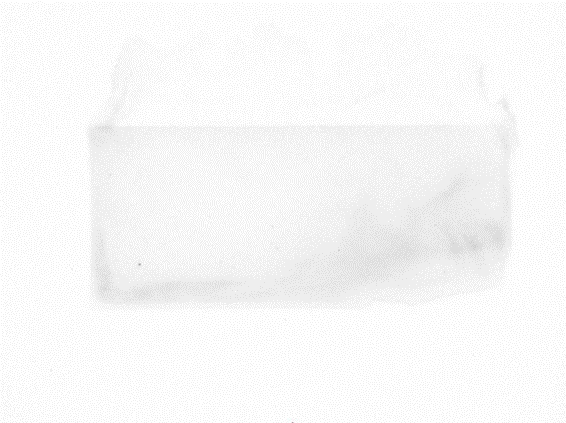
*

*Figure 5 Lamin*

*
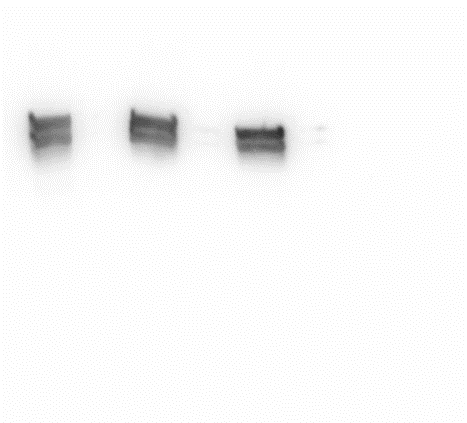
*

*Figure 5 phRIP1*

*
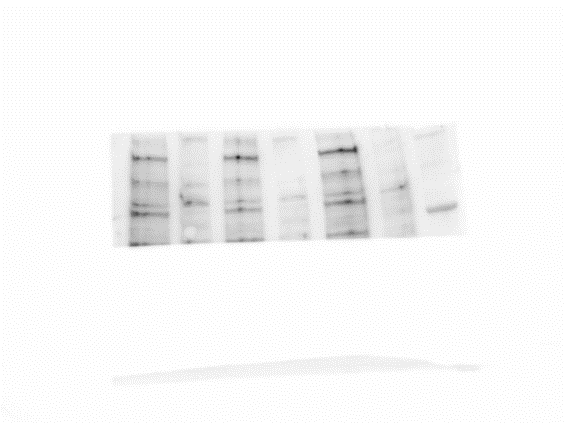
*

*Figure 5 RIP1*

*
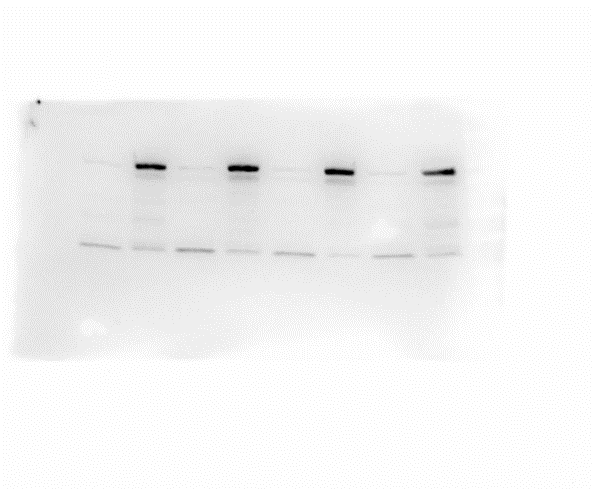
*

*Figure 5 TGF-b*

*
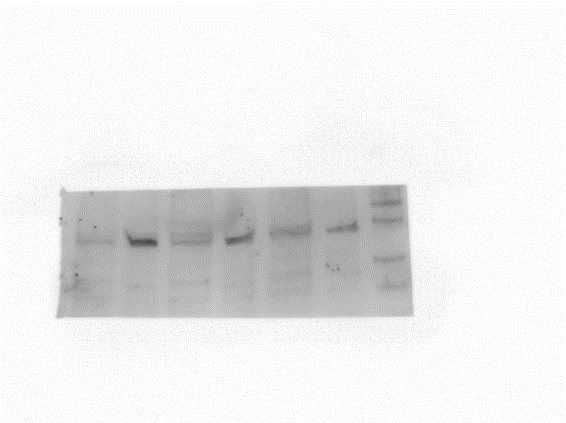
*

*Figure 6 ELOVL6*

*
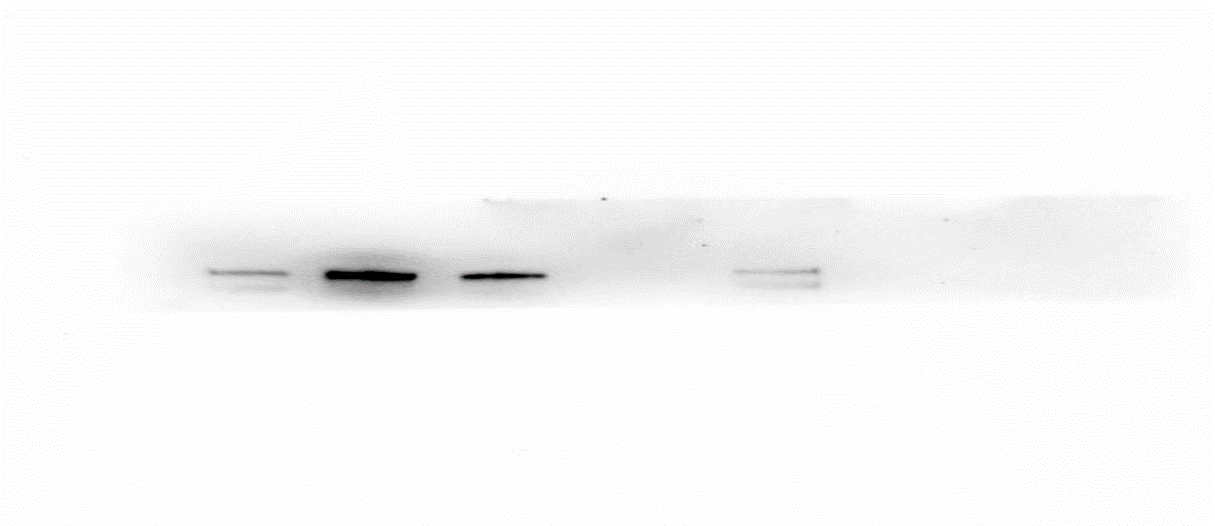
*

*Figure 6 SCD1*

*
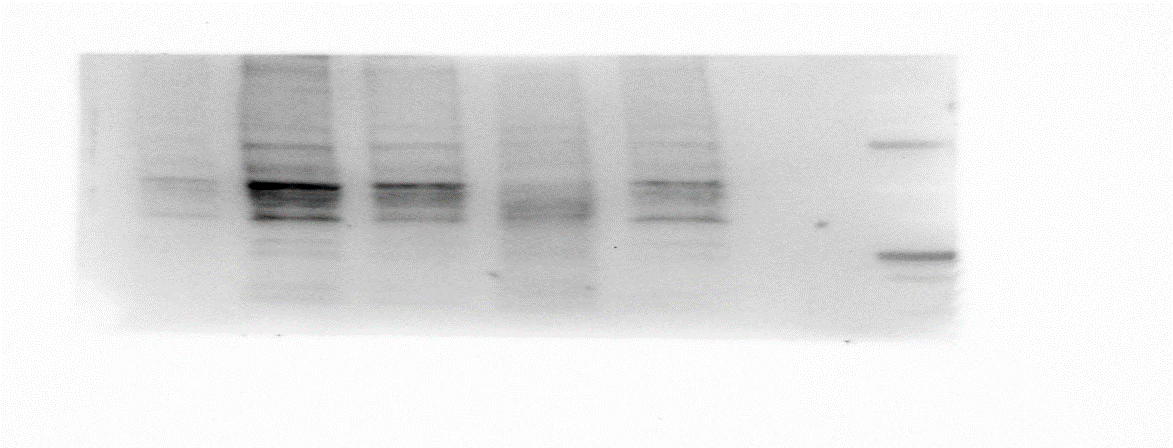
*

*Figure 6 RIP1*

*
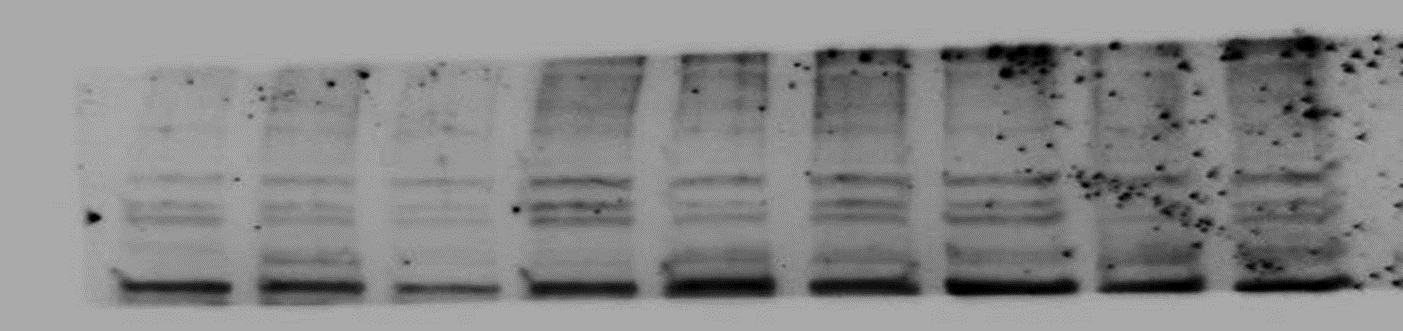
*

*Figure 6 MLKL*

*
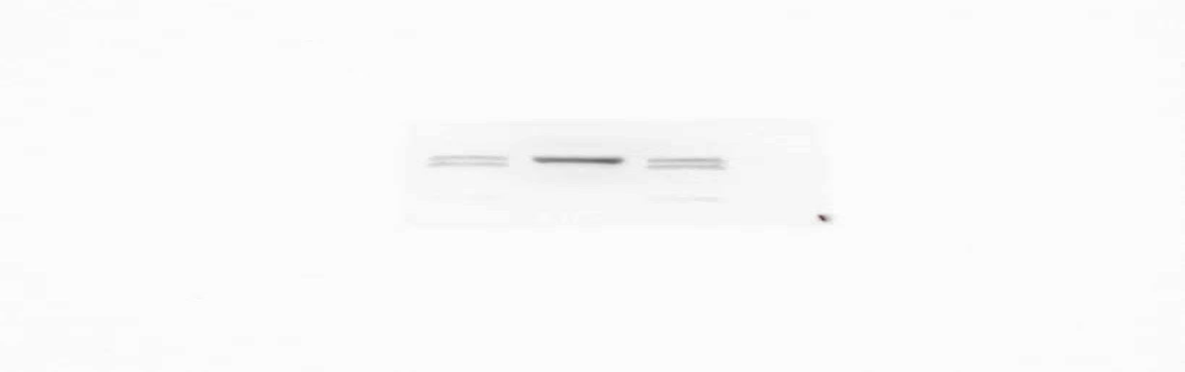
*

*Figure 6 TNF-a*

*
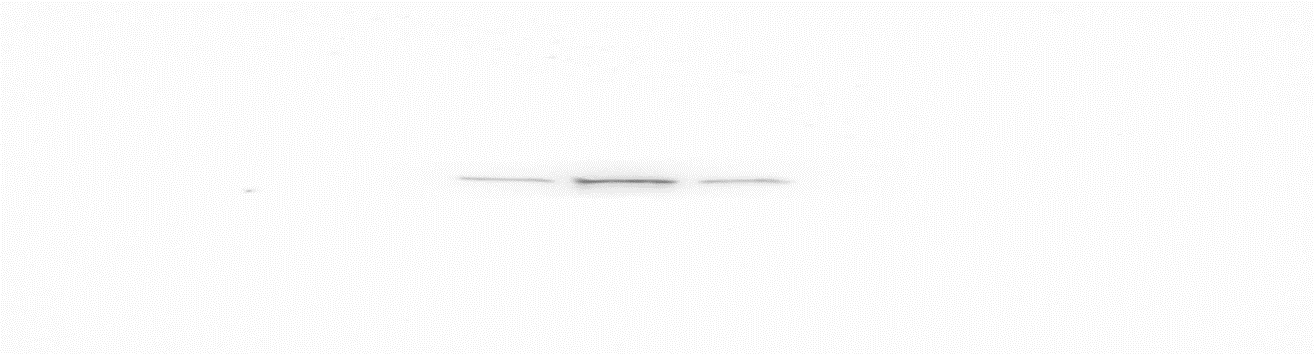
*

*Figure 6 Caspase 1*

*
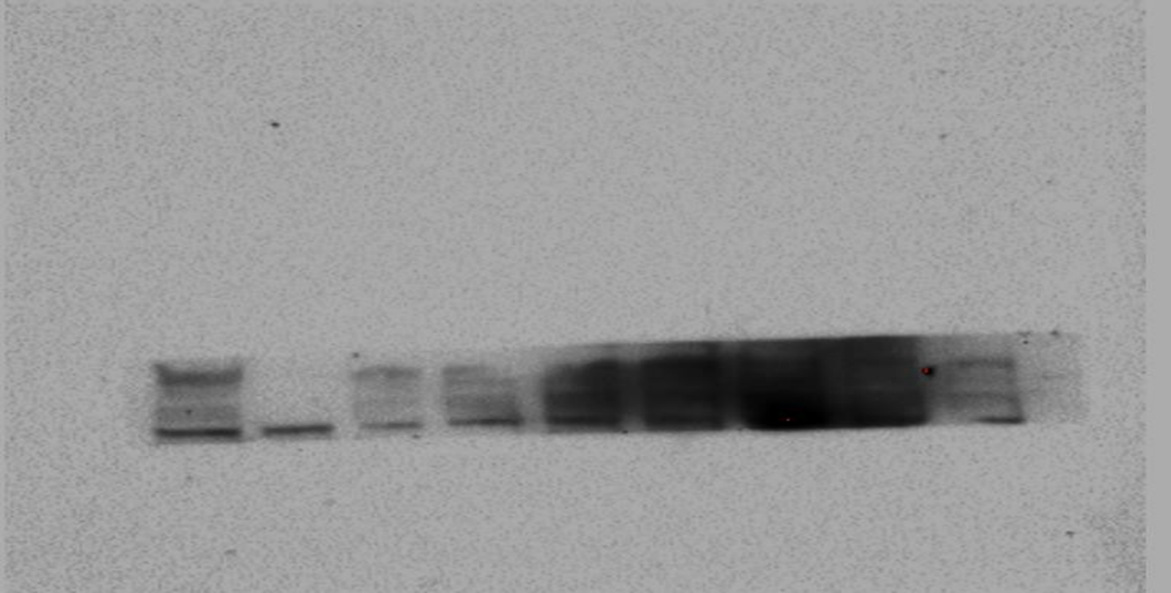
*

*Figure 6 GAPDH*

*
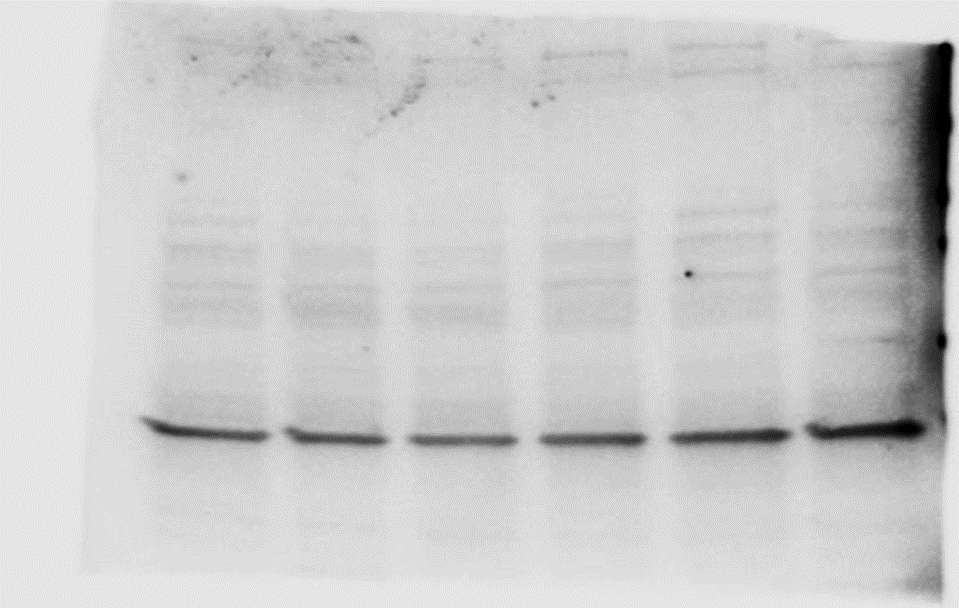
*

*Figure 7 phMLKL*

*
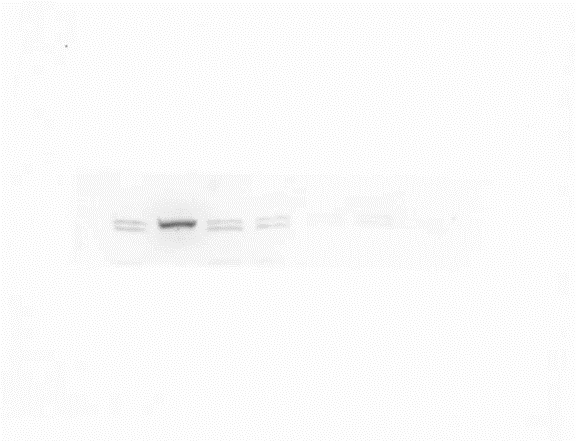
*

*Figure 7 phRIP1*

*
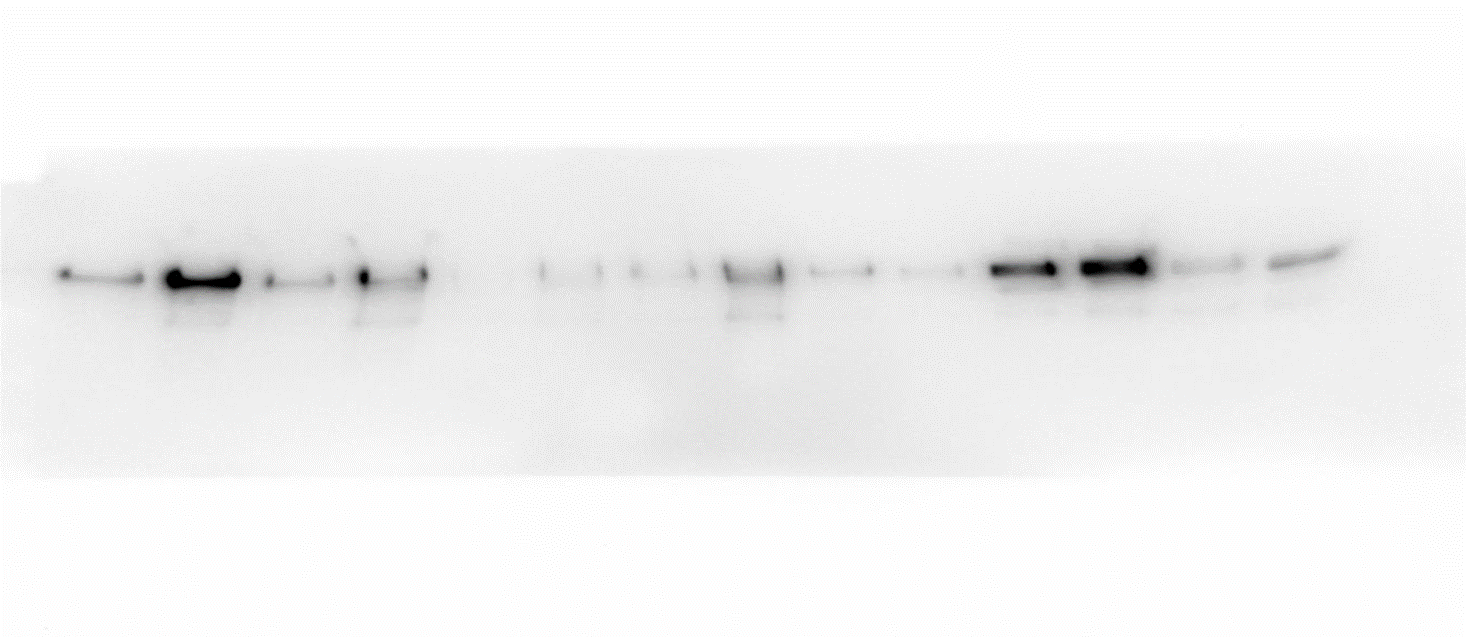
*

*Figure 7 ELOVL6*

*
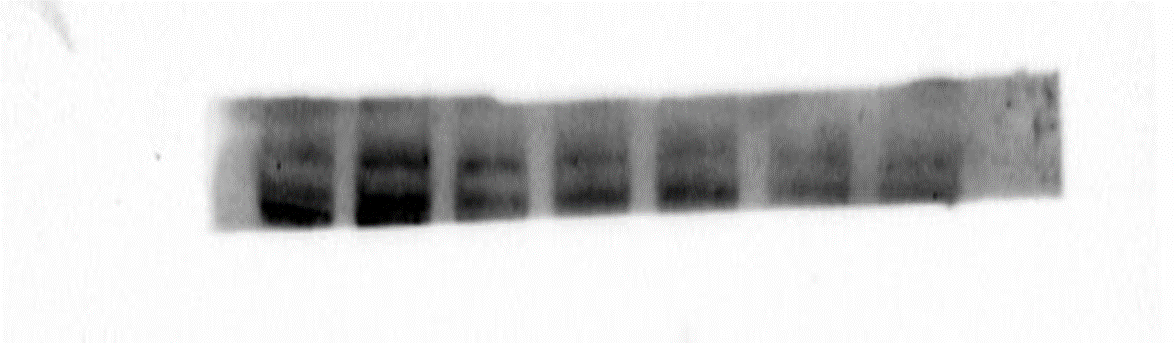
*

*Figure 7 TNF-a*

*
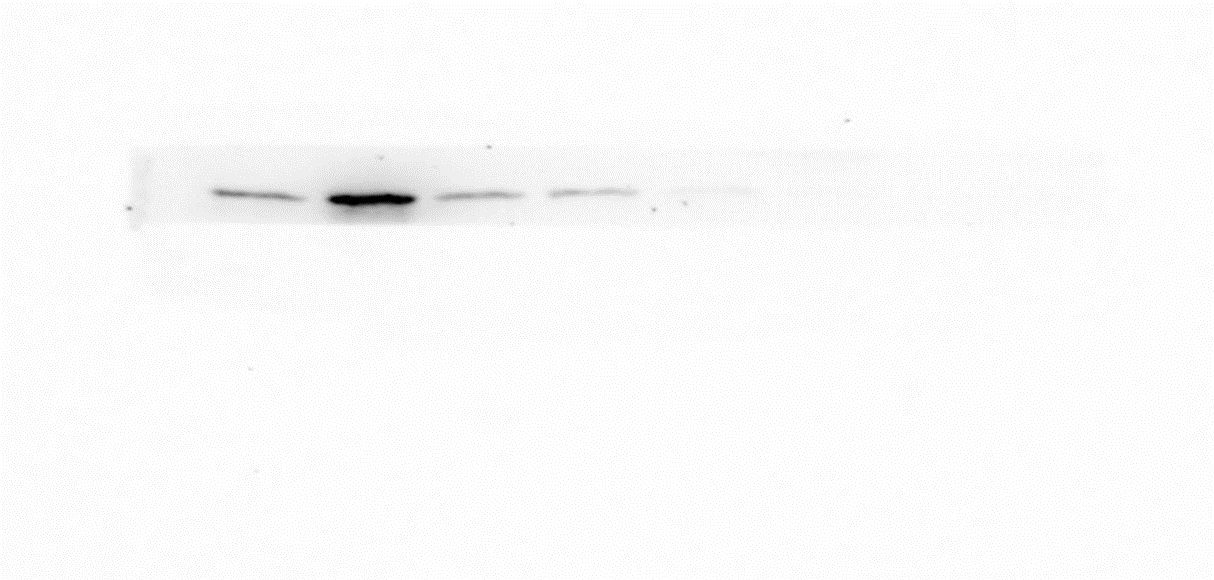
*

*Figure 7 GAPDH*

*
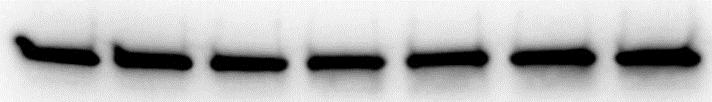
*

*Figure 7 RIP1*

*
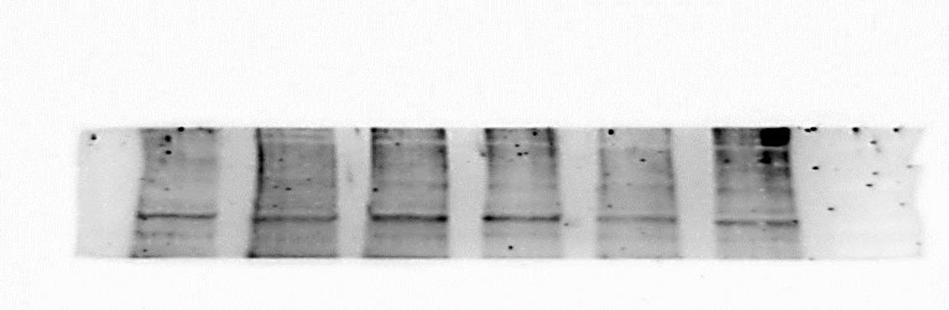
*

*Figure 7 phRIP1*

*
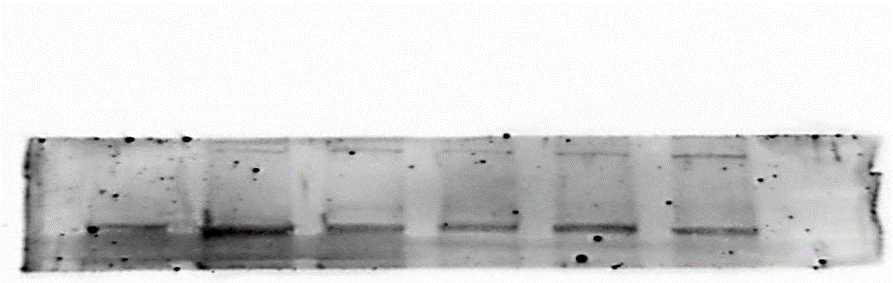
*

*Figure 7 phMLKL*

*
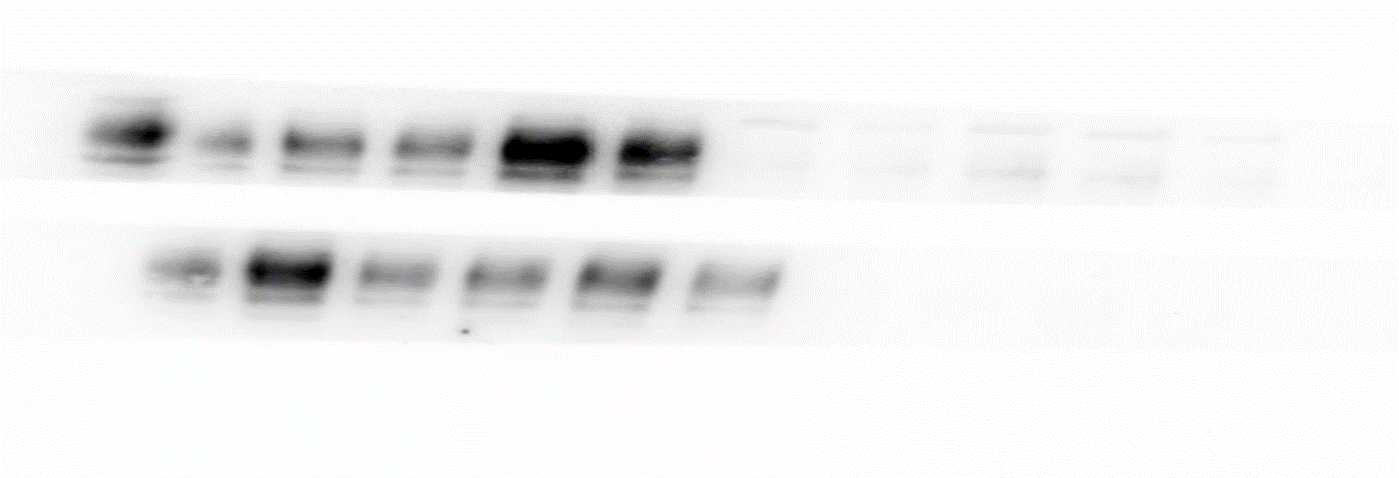
*

*Figure 2SE*

*
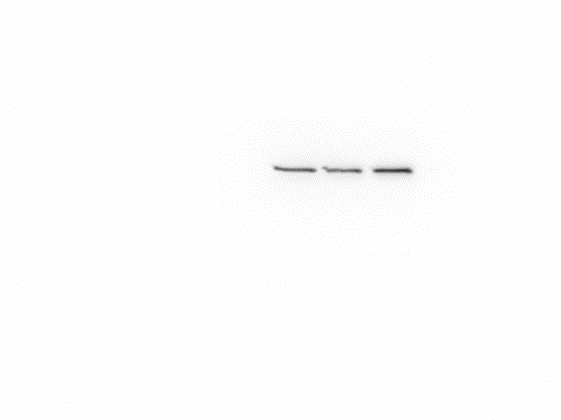
*

*
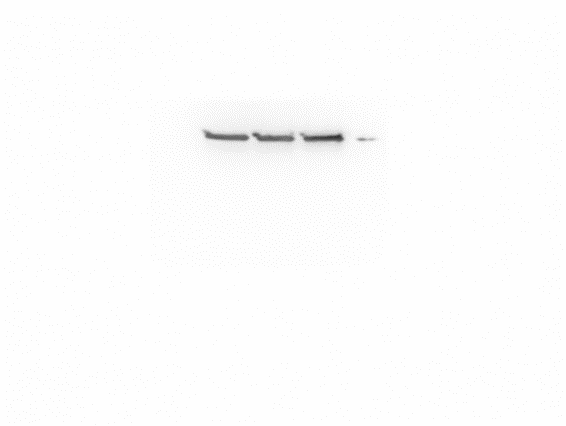
*

*Figure 3S ELOVL6*

*
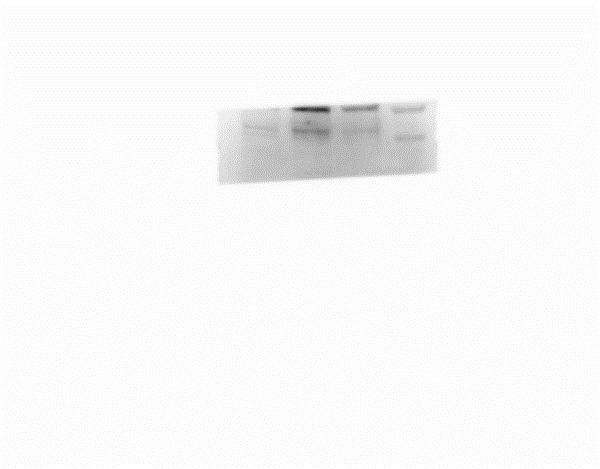
*

*Figure 3S SCD1*

*
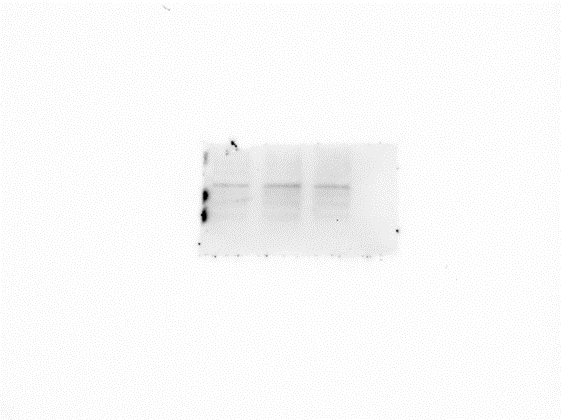
*

*Figure 3S RIP1*

*
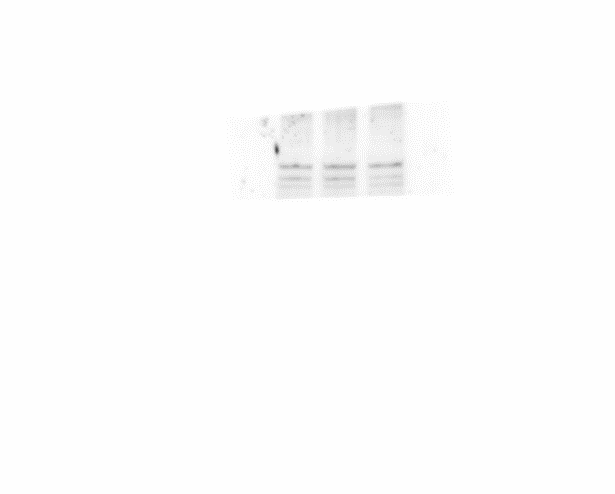
*

*Figure 3S MLKL*

*
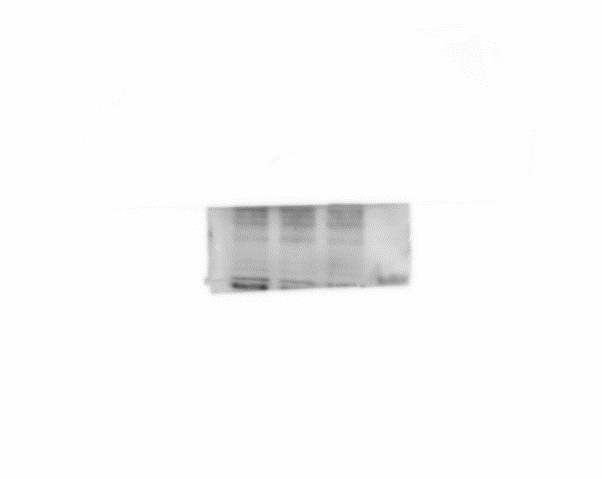
*

*Figure 3S TNF-alpha*

*
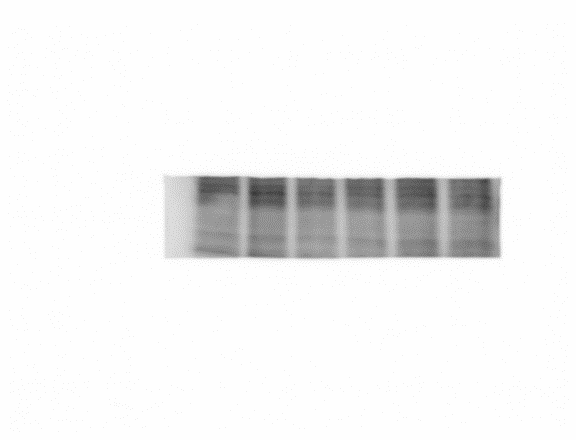
*

*Figure 3S Caspase 1*

*
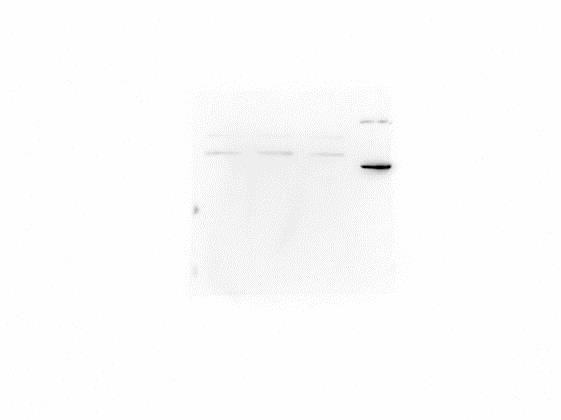
*

*Figure 3S GAPDH*

*
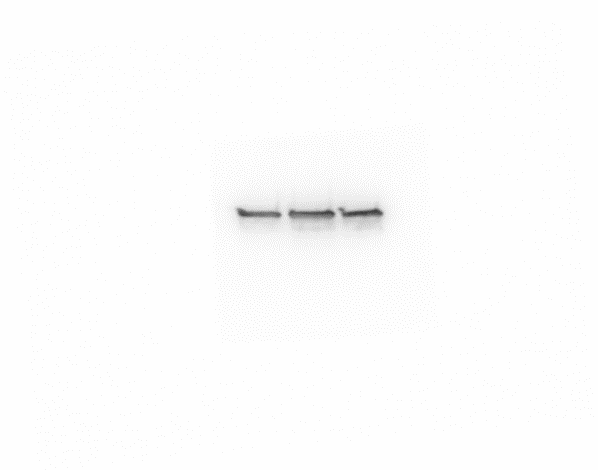
*
